# Hydrogen‐Producing Catalysts Based on Ferredoxin Scaffolds

**DOI:** 10.1002/advs.202501897

**Published:** 2025-06-17

**Authors:** Yiting She, Vera Engelbrecht, Jacek Kozuch, Ulf‐Peter Apfel, Sven T. Stripp, Anja Hemschemeier, Thomas Happe

**Affiliations:** ^1^ Photobiotechnology group, Faculty of Biology and Biotechnology Ruhr University Bochum Universitätsstrasse 150 44801 Bochum Germany; ^2^ Department of Physics Experimental Molecular Biophysics Free University of Berlin Arnimallee 14 14195 Berlin Germany; ^3^ Activation of Small Molecules/Technical Electrochemistry Faculty of Chemistry and Biochemistry Ruhr University Bochum Universitätsstraße 150 44801 Bochum Germany; ^4^ Fraunhofer UMSICHT Osterfelder Straße 3 46047 Oberhausen Germany; ^5^ Institute of Chemistry, Spectroscopy & Biocatalysis University of Potsdam Karl‐Liebknecht‐ Straße 24–25 14476 Potsdam Germany

**Keywords:** artificial metalloenzymes, cofactor, ferredoxin, hydrogenase, photocatalytic hydrogen production

## Abstract

Current attempts to transform our fossil fuel‐based society into a sustainable one involve learning from and employing the biochemistry of nature. The process of photosynthesis is exemplary for utilizing sunlight as a regenerative energy source. Enzymes like hydrogenases, which reduce protons to molecular hydrogen (H_2_) under ambient conditions, are model biocatalysts for generating sustainable, clean fuels. In green algae, photosynthesis and hydrogenases are coupled through ferredoxin, a small electron transfer protein. Here, it is shown that several plant‐type ferredoxins can interact with a chemically synthesized active site cofactor analog of [FeFe]‐hydrogenases in a way that allows comparably high H_2_ evolution rates. UV–vis and Fourier‐transform infrared spectroscopy indicate that the natural [2Fe‐2S] clusters of the ferredoxin hosts must be absent for a functional interaction of polypeptide and cofactor mimic and that the apo‐ferredoxins shield the H_2_‐producing cofactor from the solvent. The hybrid proteins exhibited higher O_2_ tolerance than natural [FeFe]‐hydrogenases and generated H_2_ in light‐dependent cascades based on photosystem I or the chemical photosensitizer proflavine. These features and the combination of natural hosts and cofactors might contribute to establishing sustainable light‐dependent H_2_ production systems.

## Graphical abstract

Selected plant‐type ferredoxins that lack their natural [2Fe‐2S] clusters functionally bind a hydrogenase active site cofactor and act as hydrogenases themselves. In combination with photosystem I, the light‐dependent H₂ evolution almost matches the H₂ production rates of the natural system.

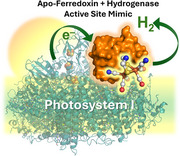

## 1 Introduction

**Figure 1:**
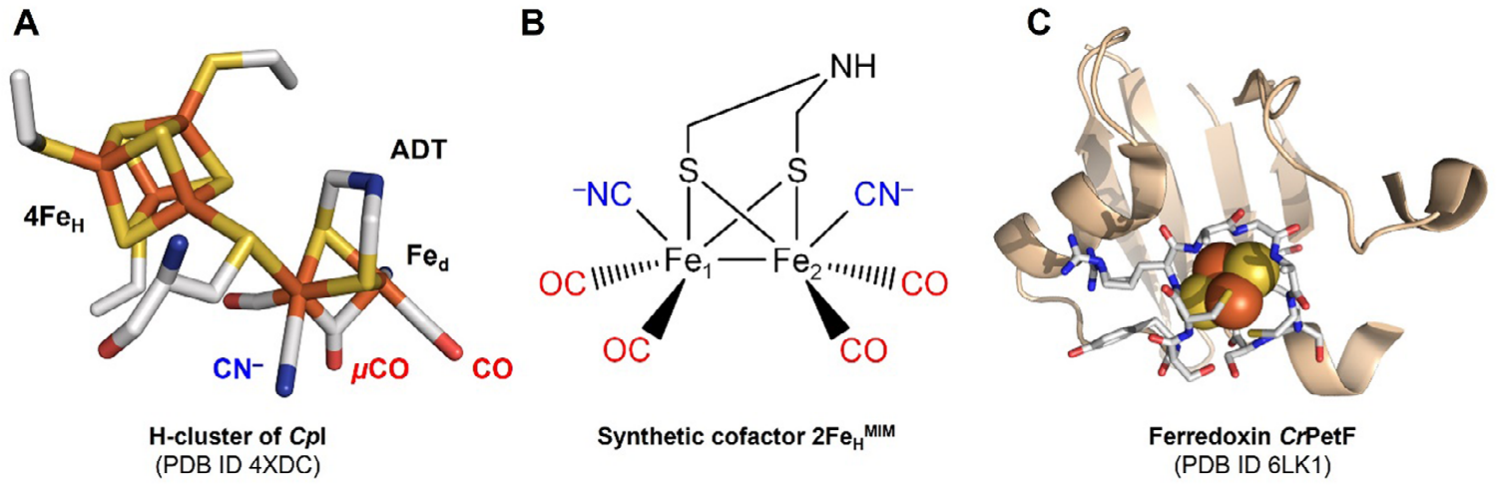
Structures of the active site cofactor of [FeFe]‐hydrogenases, the diiron site mimic employed here, and of *C. reinhardtii* PetF (*Cr*PetF). The figure depicts structures of molecules central to this study, namely A) the H‐cluster, the metal cofactor of [FeFe]‐hydrogenases (from the structure deposited at the Protein Data Bank (PDB) under ID 4XDC), B) the synthetic diiron site analog employed here, which we term 2Fe_H_^MIM^,^[^ ^9^ ^]^ and C) *C. reinhardtii* wild type PetF (PDB ID 6LK1). The protein backbone in C is depicted as a wheat cartoon. Spheres represent the [2Fe‐2S] cluster, and the loop region surrounding the [2Fe‐2S] cluster, YSCRAGACSSCAG, is depicted as sticks. A–C) The figures were created in PyMOL with the following color code: white: carbon, blue: nitrogen, red: oxygen, yellow: sulfur, orange: iron.

[FeFe]‐hydrogenases, which belong to the large metalloenzyme family of hydrogenases, play an important ecological role in that they reversibly catalyze the conversion of electrons, protons, and molecular hydrogen (H₂).^[^ ^1^, ^2^ ^]^ Members of this class of hydrogenases are widely distributed in prokaryotes and unicellular eukaryotes, where they often have a role in disposing of excess reducing equivalents from cellular metabolism.^[^ ^3^ ^]^ [FeFe]‐hydrogenases harbor a sophisticated hexanuclear iron complex, termed H‐cluster (**Figure** **1**A), and catalyze the reduction of protons to H_2_ with high rates.^[^ ^2^, ^4^, ^5^ ^]^ The H‐cluster is composed of a [4Fe‐4S] cluster (4Fe_H_), to which a unique diiron cluster (2Fe_H_) is coupled through one of the coordinating cysteines.^[^ ^6^ ^]^ The two Fe ions of 2Fe_H_ are each coordinated by a CO and a CN^−^ ligand, and a third CO ligand is found in a bridging position between both Fe ions (*µ*CO, Figure 1A).^[^ ^6^, ^7^ ^]^ Catalysis occurs at the open coordination site of the Fe ion distal to 4Fe_H_ (termed Fe_d_). Additionally, an azadithiolate ligand (ADT) links both Fe ions, the amine group serving as a proton relay.^[^ ^8^ ^]^

The biological assembly of the H‐cluster requires three dedicated assembly proteins termed maturases, which, according to current knowledge, are only present in organisms that possess [FeFe]‐hydrogenases. The 4Fe_H_ cluster is synthesized by the standard Fe‐S cluster assembly machinery so that a [FeFe]‐hydrogenase that is recombinantly produced in *Escherichia coli* – a commonly employed expression host that does not naturally possess [FeFe]‐hydrogenases – is equipped with the [4Fe‐4S] subcluster in the active site niche.^[^ ^10^ ^]^ The diiron site, however, is built by the dedicated maturases HydE, HydF, and HydG that, with contributions from the glycine cleavage system,^[^ ^11^ ^]^ assemble 2Fe_H_ in a stepwise fashion from iron, amino acids, and ammonium. HydF finally transfers it to a [FeFe]‐hydrogenase precursor already containing the 4Fe_H_ cluster.^[^ ^12^ ^]^ Note that the latter hydrogenase form is commonly, if not absolutely accurately, referred to as “apo” [FeFe]‐hydrogenase, and we use this nomenclature here, too.

The high catalytic efficiency of [FeFe]‐hydrogenases has attracted much attention for potential biotechnological applications, particularly in the development of biofuel cells and biohydrogen production.^[^ ^13^, ^14^ ^]^ However, these enzymes have not been implemented in industrial‐scale applications yet. One drawback of [FeFe]‐hydrogenases is the pronounced sensitivity against molecular oxygen (O_2_) of most members studied to date, in that binding of O_2_ at the diiron moiety leads to the degeneration of the H‐cluster.^[^ ^15^, ^16^, ^17^ ^]^ O_2_‐stable [FeFe]‐hydrogenases were identified a couple of years ago,^[^ ^18^ ^]^ and although they promise much easier handling of the biocatalysts, for example in industrial‐scale recombinant production and purification,^[^ ^19^, ^20^ ^]^ these enzymes are hardly active under oxic conditions.^[^ ^18^, ^21^, ^22^ ^]^ The comparably large molecular weight of [FeFe]‐hydrogenases can be another drawback. When these enzymes are to be coupled to surfaces such as electrodes, their size – which is about 50 kDa in the case of the smallest [FeFe]‐hydrogenases known, which are found in eukaryotic microalgae^[^ ^23^ ^]^ – limits the number of active sites per area. Smaller biocatalysts, based on smaller proteins or polymers, would also be more amendable to targeted manipulations.^[^ ^24^ ^]^

The interest in sustainable H_2_ production technologies has therefore spurred the development of artificial hydrogenases. Designing metalloenzymes is a challenge because catalysis is affected not only by the first, but also by the second coordination spheres and beyond.^[^ ^25^, ^26^, ^27^ ^]^ Most of the artificial hydrogenases designed and tested to date are based on iron, nickel, and cobalt. Metal coordination environments very different from the natural situation have been generated and shown to be catalytically competent, but suffer from low turnover frequencies (TOFs) and/or the requirement of large overpotentials.^[^ ^24^, ^28^ ^]^

In the case of [FeFe]‐hydrogenases, many structural and functional mimics of the diiron subsite of the H‐cluster have been chemically synthesized,^[^ ^29^, ^30^ ^]^ and several of these have been employed in artificial systems. For example, a diiron complex ([(µ‐S)_2_Fe_2_(CO)_6_]) with a tethered maleimide group was incorporated into the apo‐form of the naturally heme‐binding nitrobindin protein through the linkage of maleimide to a cysteine residue.^[^ ^31^ ^]^ The 2Fe_H_ analog Fe_2_[µ‐(SCH_2_)_2_NH](CN)_2_(CO)_4_^2−^ (termed 2Fe_H_^MIM^ herein) is almost identical to the natural diiron subcluster, except that it features four terminal CO ligands (Figure 1B).^[^ ^9^ ^]^ This mimic has turned out to be of particular value for [FeFe]‐hydrogenase research. It can be loaded on the maturase HydF, which is then able to activate [FeFe]‐hydrogenases containing 4Fe_H_.^[^ ^32^ ^]^ Moreover, 2Fe_H_^MIM^ alone can be mixed with an apo [FeFe]‐hydrogenase and thereupon spontaneously attaches to 4Fe_H_, losing one CO ligand during the process and forming an active H‐cluster.^[^ ^33^, ^34^ ^]^ The establishment of this artificial maturation protocol has not only contributed to research on natural maturation as well as catalysis of [FeFe]‐hydrogenases,^[^ ^12^, ^35^ ^]^ but opened up new opportunities for developing artificial hydrogenases. For example, variants of 2Fe_H_^MIM^ – containing different dithiolate ligands, metal ions, or metal ligands – can be attached to the reconstituted [4Fe‐4S] cluster of HydF from *Thermosipho melanesiensis*, which subsequently shows catalytic H_2_ production activity.^[^ ^36^, ^37^ ^]^

Environmentally sustainable H_2_ production systems require electron sources that should be sustainable themselves. In unicellular green algae, H_2_ production can be coupled to photosynthesis under certain stress conditions that result in intracellular hypoxia and the biosynthesis of [FeFe]‐hydrogenases.^[^ ^23^, ^38^ ^]^ The microalgal hydrogenases, located in the chloroplast, receive electrons from photosynthetic ferredoxin (PetF; also termed ferredoxin 1 (FDX1)), which itself is reduced by photosystem I (PSI).^[^ ^39^, ^40^ ^]^ This natural concept of employing light energy to generate low‐potential electrons for H_2_ production has long been sought to be transferred to applied systems. [FeFe]‐hydrogenases were coupled to PSI^[^ ^22^, ^41^, ^42^ ^]^ or to chemical photosensitizers.^[^ ^43^ ^]^ Other groups employed H_2_‐generating chemical moieties such as cobaloxime or nickel diphosphine in combination with PSI, which indeed resulted in light‐driven H_2_ production.^[^ ^44^, ^45^ ^]^ Silver et al. (2013) employed another strategy to target the chemical catalyst to PSI, namely by incorporating it into flavodoxin, another biological electron acceptor of PSI. They replaced the natural cofactor, flavin mononucleotide (FMN), with the nickel diphosphine catalyst, and the hybrid protein generated H_2_ light‐dependently when added to PSI.^[^ ^45^ ^]^

Making use of naturally optimized redox protein cascades is a promising strategy for modular catalytic cascades that may involve H_2_ generation. PetF is a small protein (94 amino acids in the case of mature, i.e., chloroplast‐imported PetF of the green alga *Chlamydomonas reinhardtii* ^[^ ^46^ ^]^) and belongs to the plant‐type ferredoxins that harbor single [2Fe‐2S] clusters (Figure 1C). It assumes an essential role as central electron hub in oxygenic photosynthesis by accepting electrons from PSI and delivering these to multiple redox partners, e.g., ferredoxin:nicotinamide adenine dinucleotide phosphate (NADP^+^) reductase (FNR).^[^ ^47^, ^48^ ^]^ Plant‐type ferredoxins have already been implemented in semi‐artificial H_2_‐generating assemblies. *Spinacia oleracea* (spinach) ferredoxin was employed as a scaffold for constructing a biohybrid by combining it with a ruthenium‐based photosensitizer and a cobaloxime H_2_‐generating catalyst. With the [2Fe‐2S] cluster serving as an electron relay, this biohybrid showed light‐dependent H_2_ production activity.^[^ ^49^ ^]^ Subsequently, the two chemical catalysts were separated, combining the photosensitizer with ferredoxin, and the cobaloxime with FNR, establishing a light‐dependent H_2_ production cascade based on native protein‐protein interactions.^[^ ^50^ ^]^

Here, we tested whether plant‐type ferredoxins can be employed as scaffolds for the diiron site of the H‐cluster of [FeFe]‐hydrogenases and its chemical precursor, 2Fe_H_^MIM^, respectively. Ferredoxin has the benefit of being comparably small and interacting naturally with PSI and many additional electron‐accepting enzymes.^[^ ^47^ ^]^ Moreover, 2Fe_H_^MIM^, as explained above, incorporates itself readily into [FeFe]‐hydrogenase precursors, raising hopes that it would do the same in combination with other proteins. With the impressive progress that has been made in employing the maturases HydE, HydF, and HydG for the in vitro assembly of the diiron site,^[^ ^12^ ^]^ it might furthermore be envisioned to employ a completely biological and thereby more sustainable system for generating the cofactor in vitro.

Among 13 investigated proteins, we found that the presence of certain recombinant ferredoxins enabled the 2Fe_H_^MIM^ complex to evolve H_2_ under mild conditions. The rates, although lower than those of [FeFe]‐hydrogenases, were high in comparison to other artificial hydrogenases. Moreover, H_2_ generation by the ferredoxin‐2Fe_H_^MIM^ hybrids was much less sensitive to the presence of O_2_. Spectroscopic analyses suggest that the ferredoxin polypeptides provide an environment that shields the cofactor analog from the solvent. The absence of the natural [2Fe‐2S] cluster appeared to be a precondition for a catalytically competent combination of ferredoxin and 2Fe_H_^MIM^, suggesting that the active site pocket of the ferredoxins is involved in hosting the diiron site. The chemical nature of 2Fe_H_^MIM^ was not affected upon incorporation. Notably, the hybrid proteins were capable of accepting electrons from PSI, which may facilitate their integration into modular light‐driven systems.

## 2 Experimental Section

### 2.1 Materials

Unless noted otherwise, supplies were purchased from Sigma‐Aldrich/Merck (www.sigmaaldrich.com). All solutions were prepared using ultrapure water, deionized with a Milli Q Water Purification System (Merck Millipore, www.merckmillipore.com).

### 2.2 Cloning of Protein Encoding Sequences

All ferredoxins employed here were recombinantly produced in *Escherichia coli*. **Table** **1** summarizes the protein names used here, the natural host organism, accession numbers of the original protein sequences as well as the parts overproduced here, the expression vectors, and the expression strain employed (Table 1). Note that expression vectors for producing *Cr*PetF, *Cr*Fdx2, *Cr*Fdx5, *Cr*Fdx7, and *Cr*Fdx8 were cloned before, and the encoded recombinant proteins were investigated.^[^ ^39^, ^51^, ^52^ ^]^ The codon‐optimized sequences for the additional proteins analyzed here are provided in Table S1 (Supporting Information). Based on our first results, several ferredoxins were produced with amino acid exchanges in the loop region that resulted in a motif that we term the “GGV motif” here (see Results section). In the case of *C. reinhardtii* PetF (*Cr*PetF), the exchanges were generated by QuikChange PCR on the plasmid that encodes the wild‐type protein, employing the following 5′‐overlapping mismatch primers that introduced the necessary nucleotide exchanges: PetFA39G_A41V_fw: 5′‐CCGCGGTGGTGTTTGCTCCAGCTG‐3′, PetFA39G_A41V_rv: 5′‐GCAAACACCACCGCGGCAAGAGTAGGGC‐3′. In the case of four ferredoxin‐encoding sequences that were synthesized commercially, we ordered these sequences already coding for a non‐natural “GGV motif” (see asterisks in the first column of Table 1). The coding sequence for the *C. reinhardtii* [FeFe]‐hydrogenase HydA1 (*Cr*HydA1) was present in expression vector pET21b,^[^ ^53^ ^]^ that for *Thermosynechococcus elongatus* cytochrome *c*_6_ in pASK‐IBA4. The latter plasmid was kindly donated by Prof. Marc Nowaczyk (Department of Biochemistry, University of Rostock, Germany).

**Table 1:** Ferredoxins that were heterologously produced in this study. The table lists information on the ferredoxin proteins that were recombinantly produced in this study. Expression from vector pASK‐IBA7 (IBA Lifesciences; www.iba‐lifesciences.com) equips proteins with an N‐terminal Strep‐tag followed by a factor Xa cleavage site. In the case of expression vector pET21b, the plasmid‐encoded T7‐ and His_6_‐tags were omitted by cloning, and Strep‐tag encoding sequences were included in the sequences synthesized commercially.

Name used here	Organism	The accession number of wild‐type proteins^a)^ (length of protein)	Residues of annotated protein produced here	Vector	*E. coli* strain
*Cr*PetF	*Chlamydomonas reinhardtii* (unicellular alga; Chlorophyceae)	XP_0 016 92808.1 (126 aas)	33‐126	pASK‐IBA7^b)^, ^c)^	BL21 (DE3) *ΔiscR*
*Cr*PetF_GGV_				pASK‐IBA7^b)^, ^c)^	Rosetta^TM^ (DE3)
*Cr*Fdx2	*C. reinhardtii*	XP_0 016 97912.2 (121 aas)	28‐121	pASK‐IBA7^b)^, ^c)^	BL21 (DE3) *ΔiscR*
*Cr*Fdx5	*C. reinhardtii*	XP_0 016 91603.1 (130 aas)	28‐130	pASK‐IBA7^b)^, ^c)^	BL21 (DE3) *ΔiscR*
*Cr*Fdx7	*C. reinhardtii*	XP_0 017 02098.1 (133 aas)	21‐133	pASK‐IBA7^c)^, ^d)^	BL21 (DE3) *ΔiscR*
*Cr*Fdx8	*C. reinhardtii*	XP_0 017 02123.2 (197)	22‐197	pASK‐IBA7^b)^, ^c)^	BL21 (DE3) *ΔiscR*
*Cv*FdxA	*Chlorella variabilis* (unicellular alga; Trebouxiophyceae)	Protein ID 28 321 (163 aas)	71‐163	pET21b^d)^, ^e)^	BL21 (DE3) *ΔiscR*
*Mc*Fdtr	*Micractinium conductrix* (unicellular alga; Trebouxiophyceae)	PSC69244.1 (304 aas)	33‐134	pET21b^c)^, ^d)^	BL21 (DE3) *ΔiscR*
*Co*Fd	*Chlorella ohadii* (unicellular alga; Trebouxiophyceae)	KAI7839786.1 (140 aas)	30‐140	pET21b^c)^, ^d)^	BL21 (DE3) *ΔiscR*
*Ca*Fd_GGV_ ^f)^	*Capsicum annuum* (sweet pepper)	AAD02175.1 (144 aas)	50‐144	pET21b^c)^, ^d)^	BL21 (DE3) *ΔiscR*
*Cm*Fd_GGV_ ^f)^	*Cyanidioschyzon merolae* (unicellular red alga)	NP_849 098.1 (97 aas)	2‐97	pET21b^c)^, ^d)^	BL21 (DE3) *ΔiscR*
*Ap*Fd_GGV_ ^f)^	*Arthrospira platensis* (cyanobacterium)	P00246.2 (99 aas)	4‐99	pET21b^c)^, ^d)^	BL21 (DE3) *ΔiscR*
*Mm*Fd_GGV_ ^f)^	*Monoraphidium minutum* (unicellular alga; Chlorophyceae)	KAI8467501.1 (128 aas)	35‐128	pET21b^c)^, ^d)^	BL21 (DE3) *ΔiscR*

^a)^ NCBI GenPept, except *Cv*FdxA: JGI PhycoCosm, *Chlorella variabilis* NC64A v1.0;

^b)^ amplified from cDNA derived from total RNA isolated from *C. reinhardtii* strain CC‐124;

^c)^ recombinant protein was equipped with an N‐terminal Strep‐tag;

^d)^ commercial gene synthesis; coding sequence codon‐optimized for *E. coli* by Thermo Fisher Scientific (www.thermofisher.com) or Biocat (www.biocat.com);

^e)^ recombinant protein was equipped with a C‐terminal Strep‐tag; and

^f)^ sequence ordered to encode the “GGV motif”; these proteins are indicated by the subscript suffix GGV.

Abbreviations: aas: amino acids; Fdtr: truncated form of a ferredoxin.

### 2.3 Heterologous Production and Purification of Proteins

*E. coli* strain BL21(DE3) *ΔiscR* ^[^ ^54^ ^]^ was employed to recombinantly produce all ferredoxins except the *Cr*PetF variant with the GGV motif (*Cr*PetF_GGV_), which was produced in *E. coli* Rosetta (DE3). Strain BL21(DE3) *ΔiscR* was always cultivated in the presence of 40 µg × mL^−1^ kanamycin, and *E. coli* Rosetta (DE3) on 30 µg × mL^−1^ chloramphenicol. Ampicillin (100 µg × mL^−1^) was employed to select for the presence of expression plasmids. *E. coli* strain BL21(DE3) *ΔiscR* was also used to produce *Cr*HydA1 and *T. elongatus* cytochrome *c*_6_. The latter was co‐expressed with cytochrome maturation genes, present on plasmid pEC86.^[^ ^55^ ^]^ *Cr*HydA1 was produced and purified as described before.^[^ ^53^, ^56^ ^]^

For the recombinant production of *Cv*FdxA (see Table 1 for the ferredoxin abbreviations used here) and *Cr*HydA1, *E. coli* BL21(DE3) *ΔiscR* cells were grown aerobically at 37 °C in lysogeny broth (LB‐) medium supplemented with 0.1 m morpholinopropanesulfonic acid, pH 7.4, 2 mm ammonium iron‐citrate and 5 g × L^−1^ glucose until an optical density at 600 nm (OD_600_) of 0.35 – 0.5 was reached. Then, the cultures were transferred into an anoxic chamber (Coy; www.coylab.com containing an atmosphere of N_2_ : H_2_ of 98 : 2; O_2_ concentration below 35 ppm). Subsequently, 25 mm sodium fumarate, 5 mm L‐cysteine, and 0.1 mm isopropyl β‐D‐1‐thiogalactopyranoside (IPTG) were added. Expression was conducted for 16 to 18 h at room temperature. To produce *Mc*Fdtr, *Co*Fd, *Ca*Fd_GGV_, *Cm*Fd_GGV_, *Ap*Fd_GGV_ and *Mm*Fd_GGV_ (Table 1), *E. coli* BL21(DE3) *ΔiscR* cells were grown aerobically at 37 °C in LB medium supplemented with 2 mm ammonium iron‐citrate and 5 g × L^−1^ glucose until an OD_600_ of 0.6–0.9 was reached. Expression was subsequently induced with 0.1 mm IPTG, and the cultures were cultivated aerobically at 30 °C for 18 to 20 h. For the production of the *C. reinhardtii* ferredoxins, cells were grown aerobically at 37 °C in Vogel Bonner minimal medium until an OD_600_ of 0.5–0.6 was reached. To produce *Cr*PetF_GGV_, a modified Vogel Bonner minimal medium devoid of any iron source was used. Expression of all constructs present in pASK‐IBA7 was induced by adding 0.4 µg × mL^−1^ anhydrotetracycline, and expression was done for 16 to 18 h at 30 °C. For the production of cytochrome *c*_6_, cells were grown in a terrific broth medium until an OD of 0.8–1 was reached. Protein production was induced by adding 0.2 µg × mL^−1^ anhydrotetracycline and conducted for 15 to 20 h at room temperature.

After heterologous expression, *E. coli* cultures were harvested by centrifugation (20 min at 4700 *x* g and 4 °C). Purification of all proteins except cytochrome *c*_6_ was done in the anoxic chamber. Pelleted cells were resuspended in an anoxic lysis buffer (0.1 m Tris‐HCl pH 8, 10% (v/v) glycerol, 10 mg × mL^−1^ lysozyme) and treated by sonication (employing a Branson Sonifier 250; 5 cycles à 45 seconds, output level 3, 2 min breaks between cycles, solutions were kept on ice during the process). After ultracentrifugation (120 000 *x* g, 4 °C, 60 min), the supernatants were additionally cleared by passing them through 0.2 µm pore size sterile filters. Recombinant *Cr*HydA1 and ferredoxins were purified employing Strep‐Tactin Superflow high‐capacity resin (IBA Lifesciences), using 0.1 m Tris HCl, pH 8, for all equilibration and washing steps, and the same buffer supplemented with 2.5 mm desthiobiotin for elution. *T. elongatus* cytochrome *c*_6_ was purified using a Ni‐NTA column (cOmplete His‐Tag Purification Resin, Roche), also using 0.1 m Tris‐HCl, pH 8, as the buffer, which was supplemented with 2 m imidazole for elution.

Protein samples were concentrated in 0.1 m Tris‐HCl, pH 8, using Amicon Ultra centrifugal filters (Merck Millipore). Protein concentration and purity were monitored by Bradford assay (Bio‐Rad; www.bio‐rad.com), UV–vis spectroscopy (BioPhotometer D30 from Eppendorf, www.eppendorf.com), and sodium dodecyl sulfate–polyacrylamide gel electrophoresis (SDS‐PAGE). All proteins were stored at −80 °C until further use.

### 2.4 Preparation of the Chemical Analogue (2Fe_H_^MIM^) of the 2Fe_H_ Site of the H‐Cluster

Synthesis of 2Fe_H_^MIM^ ([2Fe_2_[µ‐(SCH_2_)_2_NH](CN)_2_(CO)_4_]^2−^) and the propanedithiolate variant ([2Fe_2_[µ‐(SCH_2_CH_2_CH_2_S](CN)_2_(CO)_4_]^2−^) was done following the previously published protocols.^[^ ^9^, ^17^ ^]^

### 2.5 Incubation of 2Fe_H_^MIM^ with Ferredoxins and In Vitro Maturation of [FeFe]‐Hydrogenases

All steps were carried out in the anoxic chamber described above. The purified ferredoxins were incubated with a five‐fold molar excess of 2Fe_H_^MIM^ in Tris‐HCl, pH 8, at 8 °C for 1 h. Afterward, the protein solutions were rebuffered to 0.1 m potassium phosphate buffer, pH 6.8, and purified from unbound 2Fe_H_^MIM^ by size exclusion chromatography using NAP 5 columns (GE Healthcare, www.gehealthcare.com). Samples were then concentrated using Amicon Ultra centrifugal tubes with a cut‐off of 10 kDa and stored at −80 °C until further use. Ferredoxins that were incubated with 2Fe_H_^MIM^ in that way are termed using the names indicated in Table 1 with the suffix ─2Fe_H_^MIM^ throughout the text. For in vitro maturation of *Cr*HydA1, the same protocol was followed, with the exception that all buffers were supplemented with 2 mM sodium dithionite (NaDT), and that a 30 kDa Amicon Ultra centrifugal tube was used for sample concentration.

### 2.6 In Vitro H_2_ Production Assay

In vitro, H_2_ production activity was analyzed using NaDT‐reduced methyl viologen (MV) as an electron donor. For this, 0.5–20 nmoles of ferredoxin were mixed with 10 mm MV and 100 mm NaDT in a total volume of 200 µL in 0.1 m potassium phosphate buffer, pH 6.8, in a gastight headspace vial. The vial was purged for 4 min with argon gas and then incubated for 30 min at 37 °C in a shaking water bath. Activity assays of *Cr*HydA1 were done accordingly, except that 0.016 nmoles of protein were employed in a total reaction volume of 2 mL. After the incubation, 400 µL of gas were withdrawn from the headspace vial and analyzed by gas chromatography (GC‐2010 from Shimadzu (www.shimadzu.com) equipped with a PLOT fused silica‐coated molecular sieve column, 10 m × 0.32 mm, pore size 5 Å, from Varian). Assays were done in technical triplicates and for two to three independent protein batches.

### 2.7 Air Tolerance Assays

Air tolerance of *Mc*Fdtr‐2Fe_H_^MIM^ and *Cr*PetF_GGV_‐2Fe_H_^MIM^, compared to *Cr*HydA1, was determined by incubating protein samples in the air for defined time points. Afterward, their H_2_ production activity was analyzed using the same NaDT‐ and MV‐based in vitro H_2_ production assay as described above. For each time point, 10 µL of 200 µm protein solutions in NaDT‐free 0.1 m Tris‐HCl buffer, pH 8, were removed from the anoxic tent in a 200 µL reaction tube that was left open for the indicated minutes. Afterward, 2 µL containing 0.4 nmoles of ferredoxins or, after dilution, 0.017 nmoles of *Cr*HydA1 were transferred to the standard anoxic in vitro hydrogenase activity assay and incubated as indicated above. These experiments were done using two independent protein batches, measuring at each time point in three technical replicates.

### 2.8 PSI‐ or Proflavine Dependent H_2_ Production

Assays were done similar to previously described protocols^[^ ^39^, ^57^ ^]^ and prepared in the anoxic chamber. *T. elongatus* photosystem I (*Te*PSI) was kindly donated by Marc Nowaczyk (Department of Biochemistry, University of Rostock, Germany). The reaction mixtures for PSI‐dependent H_2_ production contained 5 mM sodium ascorbate, 0.8 mm 2,6‐dichlorophenolindophenol (DCPIP), 0.1 mm NaDT, 30 µm *T. elongatus* cytochrome *c*_6_, PSI preparations equivalent to 20 µg chlorophyll, 30 µm ferredoxin or ferredoxin‐2Fe_H_^MIM^ combinations, and, when indicated, 50 nm *Cr*HydA1, in PSI reaction buffer (20 mm Tricin‐NaOH, pH 7.6, 10 mm MgCl_2_, 0.03% (w/v) β‐D‐dodecylmaltoside) in a total volume of 200 µL in gas‐tight headspace flasks. Reaction mixtures were prepared on ice in the dark, purged with argon gas to remove H_2_ from the tent atmosphere for 4 min, and then incubated in white LED light with an intensity of 1,300 W × m^−2^ for 1 h at 37 °C and shaking at 300 rpm. Reaction mixtures for proflavine‐dependent H_2_ production had a total volume of 200 µL and contained 40 mm EDTA as a sacrificial electron donor and 200 µm proflavine (acridine‐3,6‐diamine) as a photosensitizer in 100 mM potassium phosphate buffer, pH 6.8, supplemented with 0.1 mM NaDT. The assays contained 20 µm *Mc*Fdtr‐2Fe_H_^MIM^ or 30 µM *Cr*PetF_GGV_‐2Fe_H_^MIM^. Reaction mixtures were prepared on ice in the dark, purged with argon, and then incubated in white LED light for 0.5 h at 37 °C and shaking at 300 rpm. In the case of both assays, H_2_ was then quantified in the headspace by gas chromatography. For both assays, control reactions were set up by omitting PSI or proflavine. Any H_2_ that was detected in these samples was subtracted from the H_2_ amounts quantified in the actual assays. Experiments were conducted with two biological and two technical replicates.

### 2.9 Attenuated Total Reflectance Fourier Transform Infrared (ATR FTIR) Spectroscopy

A Tensor 27 Fourier‐transform infrared (FTIR) spectrometer (Bruker Optik, www.bruker.com) equipped with a narrow‐band mercury cadmium telluride (MCT) detector and a three reflections Si/ZnSe attenuated total reflectance (ATR) optical cell (DuraSampIIR, Smiths Detection) was used, kept under anoxic conditions in a Coy Laboratory glove box. Absorbance spectra from 4000–1000 cm^−1^ were recorded at 25 °C with a resolution of 2 cm^−1^, 80 kHz scanning velocity, and 1.000 interferometer scans per spectrum. *Mc*Fdtr‐2Fe_H_^MIM^ protein solution was concentrated to 650 µm in 0.1 m Tris‐HCl, pH 8.0, and a 1 µL sample was applied to the ATR crystal. Protein films were prepared by evaporation under dry N_2_ for 2–3 min, followed by rehydration under a N_2_ aerosol for 60–90 min, as described previously.^[^ ^58^ ^]^ Solutions of 2Fe_H_^MIM^ (100 g × L^−1^) in H_2_O, DMSO, and 30% DMSO in H_2_O, were analyzed in liquid form, i.e., without evaporation of solvent. Because the cofactor analog is unstable in aqueous DMSO solutions,^[^ ^59^, ^60^ ^]^ measurements were done within 10 min after the transfer to the water‐DMSO environment. For comparisons with *Mc*Fdtr‐2Fe_H_^MIM^, aqueous solutions of 2Fe_H_^MIM^ were diluted to 10 g × L^−1^. Spectra were recorded on two independent protein batches and three independent batches of synthesized 2Fe_H_^MIM^.

### 2.10 Density Functional Theory (DFT) Calculations and Normal Mode Analysis

Optimization and normal mode analysis of 2Fe_H_^MIM^ with different ligation patterns were performed using the BP86 functional^[^ ^61^, ^62^ ^]^ in Gaussian 16.^[^ ^63^ ^]^ Fe and S atoms were described using the def2‐TZVP basis set,^[^ ^64^ ^]^ whereas C, H, N, and O atoms were treated using 6–31+g** (diffuse basis set to account for negative charge)^[^ ^65^, ^66^ ^]^ as described in previous studies.^[^ ^67^ ^]^ All steps were performed either in vacuo or using a polarizable continuum model of water. Initial structures were based on ideal pyramidal coordinations of both Fe centers with CN^−^ and CO ligands in either apical or equatorial positions (all five combinations were considered, excluding enantiomers). A net charge of −2 was set to account for the formal redox state of Fe^I^ (charge of +1) of both metal centers and the charge of the two cyanides (−1) and the ADT (‐2) ligands. Finally, an anti‐ferromagnetically coupled singlet spin state was set by assigning an *alpha* and *beta* unpaired spin on the Fe centers when generating a guess of the wave function. Geometry convergence, using the absence of imaginary frequencies after normal mode analysis, and the stability of the wavefunction were confirmed. Spectra from normal mode analysis are displayed using Lorentzian band shapes with a full width at half maximum of 8 cm^−1^. Assignment of the normal modes to specific CO/CN^−^ ligands was performed by quantifying the relative amplitudes of the mass‐weighted displacement coordinates for each normal mode.

### 2.11 Detection of CO by a Hemoglobin‐Based Assay

To analyze whether CO was released upon the interaction of 2Fe_H_^MIM^ with ferredoxins, a hemoglobin assay was conducted as described previously.^[^ ^33^, ^68^ ^]^ Briefly, bovine hemoglobin (Hb; Sigma‐Aldrich/Merck) was reduced to obtain deoxy‐Hb (Hb‐Fe^II^) under anoxic conditions employing NaDT. Reduced Hb was separated from excess NaDT using a size exclusion column. UV–vis spectra as well as single‐wavelength kinetics were recorded with an Eppendorf BioSpectrometer at 22 °C in 1 mL micro UV–cuvettes. To detect the release of CO, reaction mixtures containing 14 µm reduced Hb, 20 µM 2Fe_H_^MIM^, and 6 µm *Mc*Fdtr or 2 µm apo *Cr*HydA1 in 0.1 m potassium phosphate buffer, pH 6.8, supplemented with 2 mm NaDT, were added to cuvettes and the measurement was started. The absorbance at 419 nm (i.e., the Soret band maximum of CO‐Hb) was recorded for 20 min. The assay was done employing two independent protein batches in each case.

### 2.12 UV–vis Spectroscopy

Protein solutions of 50 to 100 µm of proteins were prepared in air‐saturated 100 mm Tris‐HCl buffer, pH 8.0, to obtain the oxidized form of the ferredoxins. UV‐Vis spectroscopy was conducted at 25 °C using an Eppendorf BioSpectrometer that was present in the anoxic glove box. Spectra of two independent protein batches were recorded.

### 2.13 Statistical Analyses

All experiments were done with at least two, but mostly more biological replicates, the latter referring to different protein batches. Details are indicated for each experimental section. Activities were then calculated as means from these replicates. Standard deviations were calculated in Excel and indicated as error bars.

## 3 Results

### 3.1 H_2_ Evolution Was Observed in a *C. reinhardtii* PetF Variant Combined with 2Fe_H_^MIM^

**Figure 2:**
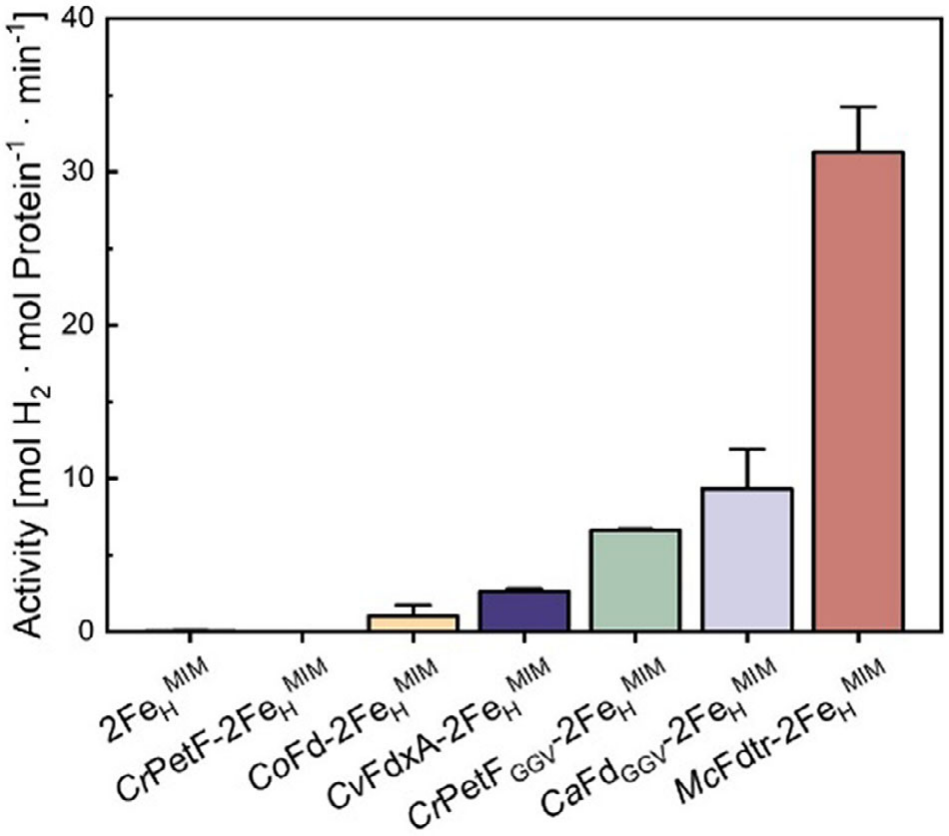
In vitro H_2_ production activities of different ferredoxins and ferredoxin variants after incubation with 2Fe_H_^MIM^. The suffix −2Fe_H_^MIM^ indicates that the respective protein was incubated with the 2Fe_H_^MIM^ complex and subsequently purified from excess 2Fe_H_^MIM^ before determining H_2_ evolution rates. The latter was done in anoxic 0.1 m potassium phosphate buffer, pH 6.8, supplemented with 10 mm methyl viologen and 100 mm sodium dithionite in a total volume of 200 µL. Samples were incubated for 30 min at 37 °C, and the H_2_ content of the headspaces was subsequently analyzed by gas chromatography. The chemical analog alone was tested as a control (indicated by “2Fe_H_^MIM^”). Data represent the mean ± standard deviation for *n* = 3 biological replicates, except CoFd–2Fe_H_^MIM^ and CaFd_GGV_–2Fe_H_^MIM^, which were measured in two biological replicates.

We aimed to generate an artificial hydrogenase based on plant‐type ferredoxins and an analog of the diiron site of [FeFe]‐hydrogenases. Our rationale was to employ ferredoxin as a promiscuous electron delivery protein, and a naturally occurring cofactor. First, employing the same experimental set‐up that is routinely used for the in vitro maturation of [FeFe]‐hydrogenases,^[^ ^33^, ^69^ ^]^ we screened several plant‐type ferredoxins from *C. reinhardtii*, namely *Cr*PetF, *Cr*Fdx2, *Cr*Fdx5, *Cr*Fdx7, and *Cr*Fdx8 for their capability to generate H_2_ after incubation with the cofactor mimic 2Fe_H_^MIM^ and subsequent purification from excess cofactor mimic (such samples will be referred to by adding the suffix −2Fe_H_^MIM^ hereafter). Additionally, we included a ferredoxin from the unicellular Trebouxiophycean alga *Chlorella variabilis* NC64A that we termed *Cv*FdxA (Table 1). While none of the analyzed *C. reinhardtii* ferredoxins generated H_2_ after incubation with 2Fe_H_^MIM^, *Cv*FdxA–2Fe_H_^MIM^ exhibited a H_2_ evolution activity of 2.6 ± 0.2 mol H_2_ × mol protein^−1^ × min^−1^ in our standard in vitro hydrogenase activity assay (**Figure** **2**).

**Figure 3:**
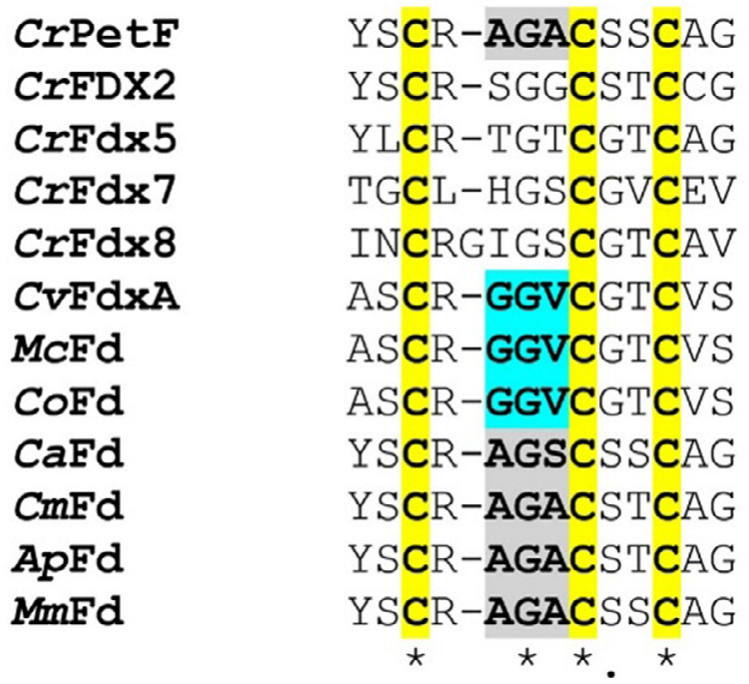
Sequence alignment of the loop regions of the ferredoxins screened for H_2_ evolution capabilities. The sequences filed under the accession numbers provided in the materials and methods section (Table 1) were aligned using Clustal Omega, keeping the input order. Afterward, the region of the loop that covers the Fe‐S center in plant‐type [2Fe‐2S]‐ferredoxins and that includes three of the cluster‐coordinating cysteine residues (bold letters labeled yellow) was manually extracted. The “GGV motif” we spotted in *C. variabilis* NC64A FdxA (*Cv*FdxA), which is also present in *M. conductrix* and *C. ohadii* ferredoxins (*Mc*Fd, *Co*Fd) is shown in bold letters and highlighted turquoise. The corresponding residues in additional ferredoxins that we exchanged to GGV are written in bold letters and highlighted gray. Fd: ferredoxin; *Cr*: *C. reinhardtii*; *Ca*: *C. anuum*; *Cm*: *C. merolae*; *Ap*: *A. platensis*; *Mm*: *M. minutum*. In the case of the *C. reinhardtii* ferredoxins, names are according to the genome annotation (Phytozome 13, *Chlamydomonas reinhardtii* CC‐4532 v6.1).

PSI acceptor proteins have been targeted as scaffolds for H_2_ evolving artificial catalysts before,^[^ ^49^, ^70^ ^]^ and photosynthetic ferredoxin PetF is the optimal electron acceptor of PSI. We, therefore, sought to equip *Cr*PetF with the capability to bind 2Fe_H_^MIM^. We assumed that the loop that connects the first three Fe‐S cluster‐binding cysteine residues and that covers the [2Fe‐2S] cluster (Figure 1C) may play a role in allowing access to the H‐cluster diiron site mimic.^[^ ^71^ ^]^ We noted a di‐glycine motif, CRGGVC, present in *Cv*FdxA, but not in *Cr*PetF (CRA_39_ GA_41_C) (**Figure** **3**). Glycine residues provide flexibility to polypeptides so that the double‐Gly motif in the loop of *Cv*FdxA, which we refer to as “GGV motif” hereafter, might allow an interaction of 2Fe_H_^MIM^ with the active site and/or the active site niche.

We introduced the corresponding amino acid exchanges into *Cr*PetF (henceforth termed *Cr*PetF_GGV_) and tested its H_2_ evolution activity after incubation with 2Fe_H_^MIM^. Notably, the hybrid protein (*Cr*PetF_GGV_‐2Fe_H_^MIM^) showed an even higher H_2_ evolution rate than the combination of *Cv*FdxA and 2Fe_H_^MIM^ and reached H_2_ production activities of 6.6 ± 0.1 mol H_2_ × mol protein^−1^ × min^−1^ (Figure 2).

### 3.2 A Truncated *Micractinium conductrix* Ferredoxin Mediated Particularly High H_2_ Evolution Rates

Although *Cr*PetF_GGV_‐2Fe_H_^MIM^ showed surprisingly high TOFs when compared to other artificial hydrogenases based on natural proteins (Table S2, Supporting Information, lists several examples), it proved unstable in many experimental attempts. We, therefore, sought to find a more robust protein host and used NCBI's protein Basic Local Alignment Search Tool (BLASTP) to identify additional ferredoxins employing *Cv*FdxA or *Cr*PetF as queries. The hits were inspected both for the GGV motif and for hosts of varying taxonomy and/or particular natural habitats. A putative [2Fe‐2S] ferredoxin from the Trebouxiophycean alga *Micractinium conductrix* was on top of the list of ferredoxins similar to *Cv*FdxA, and it contains the same loop motif as *Cv*FdxA (CRGGVCGTC) (Figure 3). The protein annotated from the genome sequence^[^ ^72^ ^]^ has an extended C‐terminus which does not contain predictable domains, and a putative chloroplast transit peptide when analyzed by PredAlgo^[^ ^73^ ^]^ and TargetP‐2.0. We thus ordered a codon‐optimized sequence for amino acids 33 to 134 of the annotated sequence (also see Table 1 in the Experimental Section) and termed the corresponding protein *Mc*Fdtr (“tr” referring to “truncated”).

We also selected ferredoxins from the Trebouxiophycean alga *Chlorella ohadii* (*Co*Fd),^[^ ^74^ ^]^ from sweet pepper (*Capsicum anuum*) (*Ca*Fd),^[^ ^75^ ^]^ from the unicellular red alga *Cyanidioschyzon merolae* (*Cm*Fd),^[^ ^76^ ^]^ one of the cyanobacterium *Arthrospira platensis* (formerly *Spirulina platensis*) (*Ap*Fd),^[^ ^77^ ^]^ and one of the Chlorophycean alga *Monoraphidium minutum* (*Mm*Fd).^[^ ^78^ ^]^ All accession numbers are provided in the Experimental Section (Table 1). The latter four sequences do not contain the GGV motif naturally (Figure 3), but the codon‐optimized sequences for their heterologous production were designed so that the respective natural amino acids were replaced by GGV. In the following, this is indicated by the subscript suffix GGV, e.g., *Ca*Fd_GGV_.

As described for the *C. reinhardtii* ferredoxins and *Cv*FdxA, these additional ferredoxins were heterologously produced in and purified from *E. coli*, and then incubated with 2Fe_H_^MIM^. After removing excess cofactor mimics, they were tested for H_2_ production capabilities. *Co*Fd–2Fe_H_^MIM^ and the *Ca*Fd_GGV_–2Fe_H_^MIM^ variant indeed showed activity, and the latter a comparably high one of 9.3 ± 2.6 mol H_2_ × mol protein^−1^ × min^−1^ (Figure 2). However, the *Mc*Fdtr–2Fe_H_^MIM^ protein was especially active, reaching an activity of 31.3 ± 2.9 mol H_2_ × mol protein^−1^ × min^−1^ (Figure 2).

As noted in the introduction, variants of 2Fe_H_^MIM^, namely diiron complexes with propanedithiolate (PDT) and propanediselenol bridging groups, were combined with the HydF maturase protein, resulting in H_2_ evolution activity, whereas the ADT compound used here (i.e., 2Fe_H_^MIM^) was not active.^[^ ^36^, ^37^, ^79^ ^]^ We therefore tested whether *Cr*PetF_GGV_ or *Mc*Fdtr would develop H_2_ production activity when combined with the PDT derivative in otherwise identical experimental setups. However, this was not the case, indicating that the ferredoxin polypeptides provide a different environment for the cofactors than HydF.

### 3.3 *Mc*Fdtr‐2Fe_H_^MIM^ and *Cr*PetF_GGV_‐2Fe_H_^MIM^ Are More O_2_‐Tolerant Than the [FeFe]‐Hydrogenase *Cr*HydA1

**Figure 4:**
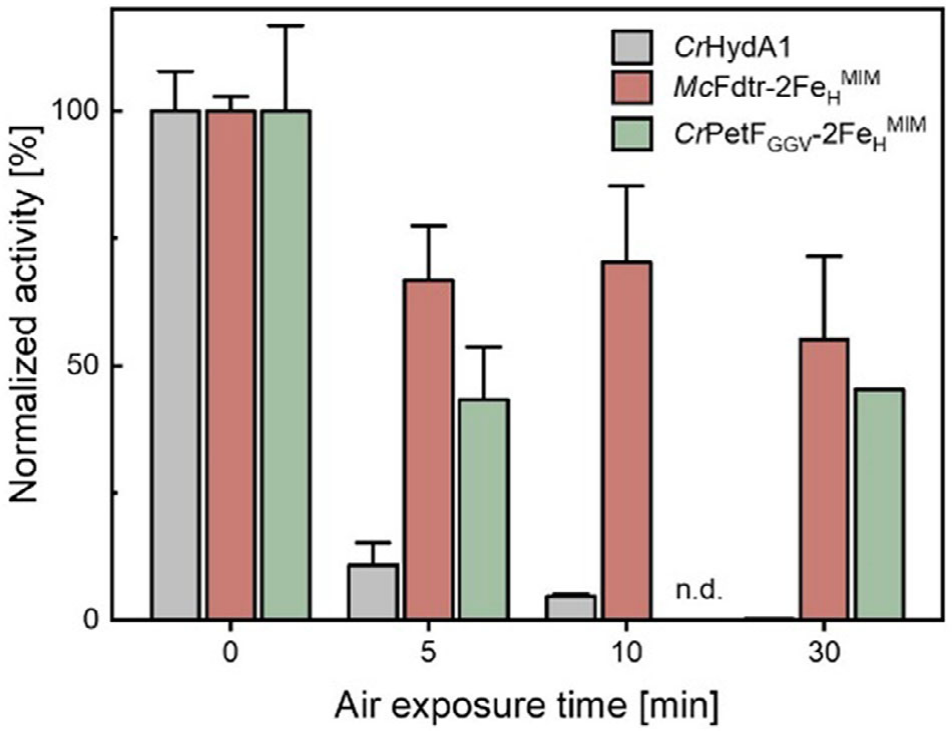
Residual H_2_ production activities of *Cr*HydA1, *Mc*Fdtr–2Fe_H_^MIM^, and *Cr*PetF–2Fe_H_^MIM^ after air exposure. Protein solutions (200 µm of each protein in 10 µL of NaDT‐free 0.1 m Tris‐HCl buffer, pH 8.0) were exposed to air for the indicated periods of time. Afterward, aliquots of the solutions were transferred to the standard anoxic in vitro hydrogenase activity assay. H₂ evolution activities relative to individual activities reached under anoxic conditions are shown, calculated from two independent biological replicates with analytical triplicates in each case. Error bars indicate the standard deviation. n.d.: not determined.

Most [FeFe]‐hydrogenases are rapidly and irreversibly inactivated by O_2_. We were curious as to whether the ferredoxin–2Fe_H_^MIM^ proteins were similarly affected by O_2_ and conducted air exposure experiments in a time series with *Mc*Fdtr‐2Fe_H_^MIM^ and *Cr*PetF_GGV_‐2Fe_H_^MIM^, employing the highly O_2_‐intolerant [FeFe]‐hydrogenase *Cr*HydA1 as a control. Protein aliquots were subjected to air exposure for 5, 10, and 30 min, and then immediately transferred to the anoxic chamber for in vitro hydrogenase activity assays. As expected, the H_2_ evolution activity of *Cr*HydA1 showed a strong decrease to 4.7 ± 0.6% of the activity it reached under anoxic conditions after being exposed to air for 10 min (**Figure** **4**). *Cr*PetF‐2Fe_H_^MIM^ retained about 45% of its activity after 30 min of air exposure, while *Mc*Fdtr‐2Fe_H_^MIM^ showed a residual activity of 55 ± 16% at the same time point (Figure 4).

### 3.4 ATR FTIR Spectroscopy Suggests That the *Mc*Fdtr Protein Shields 2Fe_H_^MIM^ from the Solvent

The results described above indicated that several of the ferredoxin proteins tested here provide some kind of coordination environment that enables 2Fe_H_^MIM^, which itself is catalytically inactive in the assays we employed (Figure 2), to reduce protons. Additionally, the air exposure experiments suggested that the ferredoxin protein shields 2Fe_H_^MIM^ from O_2_, or that the cofactor does not react with O_2_ in the same way as the H‐cluster within [FeFe]‐hydrogenases.^[^ ^15^, ^16^ ^]^

Infrared spectroscopy is commonly used to characterize [FeFe]‐hydrogenases. The stretching vibrations of the CO/CN^−^ iron ligands of the H‐cluster (Figure 1A,B) between 2200 and 1700 cm^−1^ are sensitive to redox‐ and protonation states as well as cofactor geometry, and they can serve to assess the integrity of the active site cofactor or cofactor mimics.^[^ ^2^, ^5^ ^]^ Here, we investigated whether the FTIR spectrum of 2Fe_H_^MIM^ would change upon interaction with *Mc*Fdtr, as it is the case when 2Fe_H_^MIM^ is loaded onto the [FeFe]‐hydrogenase maturating protein HydF, or on apo [FeFe]‐hydrogenases.^[^ ^32^, ^33^, ^80^ ^]^ To this aim, ATR FTIR spectra of the 2Fe_H_^MIM^ complex in comparison to *Mc*Fdtr‐2Fe_H_^MIM^ and the *Mc*Fdtr protein prior to the addition of 2Fe_H_^MIM^ were recorded.

**Figure 5:**
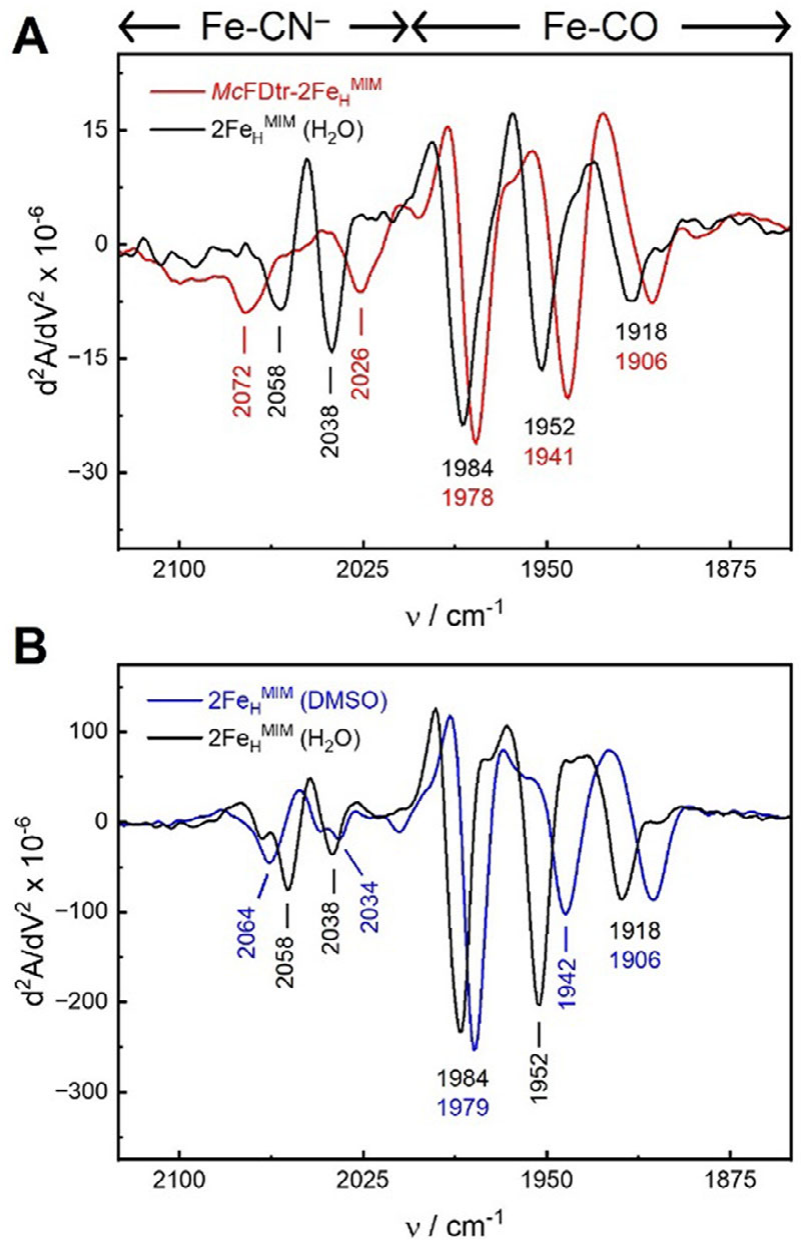
Second derivative ATR FTIR spectra of free 2Fe_H_^MIM^ and *Mc*Fdtr‐2Fe_H_^MIM^. A) Spectra were collected employing an aqueous solution of 10 g × L^−1^ (25.8 mm) 2Fe_H_^MIM^ in H_2_O (red trace) and hydrated protein films of 650 µm of *Mc*Fdtr‐2Fe_H_^MIM^ in 0.1 m Tris‐HCl, pH 8.0 (black trace). Bands < 2000 cm^−1^ are assigned to CO ligands, and bands > 2000 cm^−1^ are assigned to CN^−^ ligands.^[^ ^81^ ^]^ B) Comparison of 100 g × L^−1^ (258 mm) 2Fe_H_^MIM^ in H_2_O (red trace) and a mixture of 70% H_2_O and 30% DMSO (blue trace).

Second‐derivative ATR FTIR spectra of 2Fe_H_^MIM^ were comparable to those reported before,^[^ ^32^, ^69^ ^]^ with maxima of the broad CO/CN^−^ bands at 2074, 2024, 1967, 1922, and 1890 cm^−1^ (Figure S1A,B, Supporting Information). Density functional theory (DFT) calculations assign the bands >2000 cm^−1^ to CN^−^ and bands <2000 cm^−1^ to the coupled vibration of the terminal CO ligands (Figure S2, Tables S3 and S4, Supporting Information). Both measured and calculated spectra lack any bands below 1850 cm^−1^, indicating that the complex does not feature a bridging CO ligand (*µ*CO). The maxima noted above were only observed in dried and highly concentrated sample films, whereas the spectra of the aqueous 2Fe_H_^MIM^ solution revealed not only much less intense bands, but additionally shifted frequencies with maxima at 2058, 2038, 1984, 1952, and 1916 cm^−1^ (**Figure** **5**A; Figure S1A, Supporting Information). Due to this dependence on hydration and concentration, we aimed to compare the spectra at similar intensities and therefore employed aqueous solutions of 10 g × L^−1^ 2Fe_H_^MIM^ (corresponding to about 25.8 mm) and 650 µm solutions of *Mc*Fdtr‐2Fe_H_^MIM^.

Comparing these solutions, both samples revealed a similar CO/CN^−^ pattern, both in intensity and frequency (Figure 5A), whereas untreated *Mc*Fdtr did not exhibit any cofactor bands (Figure S3, Supporting Information). Compared to free 2Fe_H_^MIM^, however, all signals of the *Mc*Fdtr‐2Fe_H_^MIM^ sample were shifted to slightly higher frequencies by 9 ± 3 cm^−1^, except the signal of one of the CN^−^ ligands that was shifted to lower energies, from 2072 to 2058 cm^−1^ (Figure 5A). Because of this specific red‐shift, and because the water content of both samples was very similar (Figure S3, Supporting Information), band shifts due to different hydration levels of the 2Fe_H_^MIM^ compound and *Mc*Fdtr‐2Fe_H_^MIM^ can be excluded. In contrast to 2Fe_H_^MIM^, no band shifts were observed upon dehydration of *Mc*Fdtr‐2Fe_H_^MIM^ (Figure S1C,D, Supporting Information). In summary, a comparison of the FTIR spectra of *Mc*Fdtr‐2Fe_H_^MIM^ and 2Fe_H_^MIM^ suggests that 2Fe_H_^MIM^ is subject to a different environment after incubation with the *Mc*Fdtr protein than when simply present in aqueous solution.

We assumed that the changes in the FTIR spectra of 2Fe_H_^MIM^ before and after incubation with ferredoxin were due to the confinement of the cofactor that would also minimize the access of solvent. This assumption is supported by the observed band shifts upon drying (Figure S1A,B, Supporting Information). However, the associated changes in concentration might encompass effects beyond our control. An increasingly hydrophobic environment can be probed alternatively by mixing fractions of 2Fe_H_^MIM^ in H_2_O and 2Fe_H_^MIM^ in dimethyl sulfoxide (DMSO). The CO bands of 2Fe_H_^MIM^ in a mixed solvent comprised of 70% H_2_O and 30% DMSO were indeed blue‐shifted by 7 ± 3 cm^−1^ when compared to those of 2Fe_H_^MIM^ in water (Figure 5B). Similar to the dehydration series (Figure S1A,B, Supporting Information), one CN^−^ band shifted to higher energies, while the other CN^−^ band showed a red‐shift. The infrared absorbance of DMSO did not interfere with the CO/CN^−^ signatures (Figure S4A, Supporting Information). The concentration of 2Fe_H_^MIM^ was constant in both experiments so that we can rationalize the observed band shifts with a change from a protic (H_2_O) to an aprotic solvent (DMSO). The spectrum of *Mc*Fdtr‐2Fe_H_^MIM^ closely resembled that of 2Fe_H_^MIM^ in 30% DMSO (Figure 5B), suggesting that the *Mc*Fdtr protein provides a partly hydrophobic environment to the cofactor analog.

### 3.5 2Fe_H_^MIM^ Likely Interacts with the *Mc*Fdtr Protein in Its Fully Ligand‐Saturated Form

As noted in the introduction, chemically maturating apo [FeFe]‐hydrogenases with 2Fe_H_^MIM^ results in the formation of an H‐cluster. For this to happen, one iron ion of 2Fe_H_^MIM^ coordinates to a thiol group and, besides other structural rearrangements, loses its fourth CO ligand (compare Figure 1A,B), which can be followed by UV–vis absorption changes of CO binding to hemoglobin.^[^ ^33^, ^68^ ^]^ According to our FTIR and DFT analyses, 2Fe_H_^MIM^ comprises four vibrationally coupled CO ligands that give rise to three discernable CO bands in addition to the two CN^−^ bands (Figure S2, Supporting Information and Tables S3 and S4, Supporting Information). In the H‐cluster, a CO ligand bridges the two Fe ions of the diiron site and gives rise to a signal between 1860 and 1790 cm^−1^ in all catalytically relevant states (Figure S4B, Supporting Information).^[^ ^82^, ^83^, ^84^ ^]^ As neither 2Fe_H_^MIM^ nor the *Mc*Fdtr‐2Fe_H_^MIM^ hybrid gave rise to a signal in this region, we conclude that the 2Fe_H_^MIM^ complex kept its original structure with two CN^−^ and four CO ligands. We tested this employing the hemoglobin‐based spectroscopic assay mentioned above and observed that the incubation of the *Mc*Fdtr protein with the chemical mimic did not result in a significant release of CO when compared to the maturation of apo *Cr*HydA1, although the measurable CO levels were moderately higher when compared to the 2Fe_H_^MIM^ complex alone (Figure S5, Supporting Information).

### 3.6 Ferredoxins That Enabled H_2_ Evolution Had a Low [2Fe‐2S] Cluster Occupancy

Because the ATR FTIR analyses suggested that the 2Fe_H_^MIM^ complex might be shielded from the solvent by the *Mc*Fdtr polypeptide, we assumed that it could interact with the active site niche of the ferredoxin proteins that allowed H_2_ production. We, therefore, employed UV‐Vis spectroscopy to inspect the different ferredoxins with regard to their [2Fe‐2S] cluster occupancy directly after purification from *E. coli*, i.e., before they were incubated with 2Fe_H_^MIM^.

**Figure 6:**
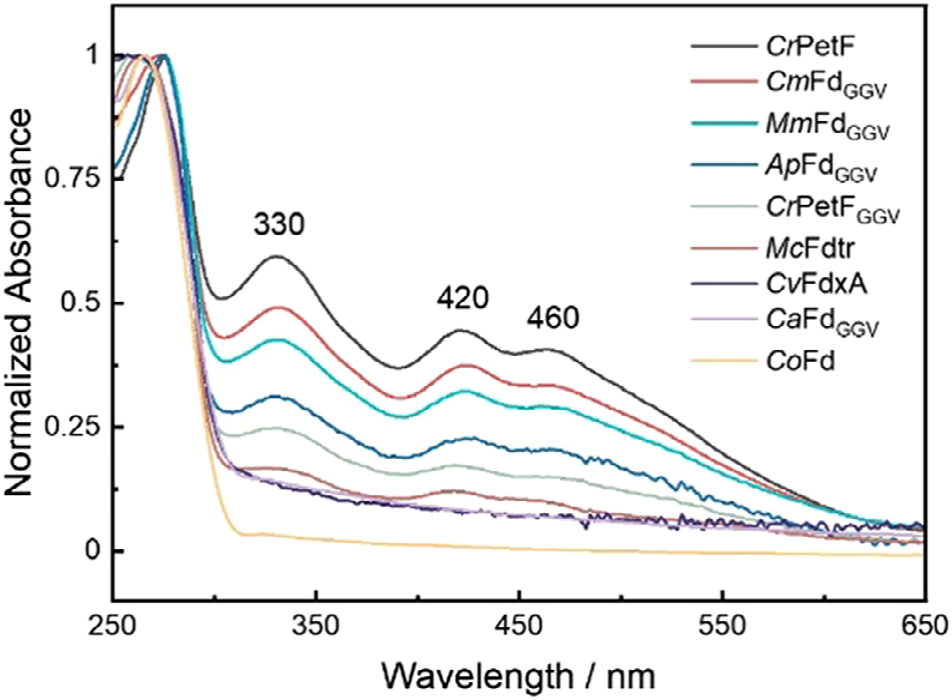
UV–vis spectroscopy shows different [2Fe‐2S] cluster occupancies of the recombinant ferredoxins. Normalized UV–vis spectra of the heterologously produced ferredoxins after their purification from the *E. coli* host, i.e., before incubating them with 2Fe_H_^MIM^. All recombinant ferredoxins were dissolved in air‐saturated 0.1 m Tris‐HCl, pH 8.0, to a concentration of 50–100 µm. UV–vis spectra were recorded at a temperature of 22 °C in a photometer located in the anoxic glove box. The data series are arranged in the order of the strength of absorption of the [2Fe‐2S] clusters, and abbreviations are as follows: Wild‐type forms of *C. reinhardtii* PetF, *C. variabilis* NC64A FdxA and *C. ohadii* ferredoxin (*Cr*PetF, *Cv*FdxA and *Co*Fd), truncated form of *M. conductrix* ferredoxin (*Mc*Fdtr), “GGV variants” of the ferredoxins from *C. reinhardtii* PetF (*Cr*PetF_GGV_), *C. merolae* (*Cm*Fd_GGV_), *M. minutum* (*Mm*Fd_GGV_), *A. platensis* (*Ap*Fd_GGV_) and *C. anuum* (*Ca*Fd_GGV_). The spectra were normalized to the absorbance maximum of the polypeptide between 260 and 276 nm. UV–vis spectra were recorded from two independent protein batches each, and one representative spectrum is shown for each ferredoxin.

UV–vis spectra of oxidized plant‐type ferredoxins usually show charge‐transfer bands of the [2Fe‐2S] cluster at 330, 420, and 460 nm, with the polypeptide maximum at 276 nm.^[^ ^85^, ^86^ ^]^ These spectra were indeed well‐resolved in several ferredoxin preparations (**Figure** **6**). In contrast, all ferredoxins that showed H_2_ evolution capability after incubation with the 2Fe_H_^MIM^ cofactor mimic, including *Cv*FdxA and *Cr*PetF_GGV_, did not or hardly show the characteristic maxima of the [2Fe‐2S] cluster (Figure 6). Additionally, we noted that the maxima of the polypeptide absorption in the UV region correlated with the absence or presence of the typical [2Fe‐2S] cluster signals: All ferredoxins that showed no or only weak maxima for Fe‐S clusters had a maximum below 266 nm (*Mc*Fdtr, *Co*Fd, and *Ca*Fd_GGV_: 266 nm, *Cv*FdxA: 263 nm, *Cr*PetF_GGV_: 260 nm), whereas all proteins with [2Fe‐2S] clusters showed maxima at 276 nm (Figure 6).

### 3.7 Light‐Driven H_2_ Production by 2Fe_H_^MIM^‐Loaded Ferredoxins

In several unicellular algae such as *C. reinhardtii*, light‐dependent H_2_ production by the natural hydrogenase system depends on electron transfer from PSI via PetF to the hydrogenase.^[^ ^23^, ^38^ ^]^ We wondered whether the ferredoxin‐2Fe_H_^MIM^ forms would still behave “ferredoxin‐like” in this regard. Therefore, we examined the capability of *Cr*PetF_GGV_‐2Fe_H_^MIM^ and *Mc*Fdtr‐2Fe_H_^MIM^ to accept electrons from PSI in an in vitro system containing *T. elongatus* PSI (*Te*PSI), cytochrome *c*_6_, and the sacrificial electron donor ascorbate that we had used before to study *Cr*PetF‐dependent H_2_ production.^[^ ^39^, ^57^ ^]^ PSI‐ and *Cr*PetF‐dependent H_2_ evolution by *Cr*HydA1 served as control and reached rates of 17.5 ± 1.8 nmol H_2_ × µg Chl^−1^ × h^−1^. In the same set‐up but replacing *Cr*PetF and *Cr*HydA1 by *Mc*Fdtr‐2Fe_H_^MIM^ or *Cr*PetF_GGV_‐2Fe_H_^MIM^ alone, H_2_ evolution rates of 0.28 ± 0.08 nmol H_2_ × µg Chl^−1^ × h^−1^ and 5.34 ± 3.19 nmol H_2_ × µg Chl^−1^ × h^−1^ were determined, respectively. In order to exclude the influence of the potentially low affinity between the *M. conductrix* ferredoxin, whose natural function is unknown, and the PSI complex, we tested an alternative system by replacing PSI by the synthetic photosensitizer proflavine, using EDTA as a sacrificial electron donor as was described before.^[^ ^57^ ^]^ In this system, *Mc*Fdtr‐2Fe_H_^MIM^ and *Cr*PetF_GGV_‐2Fe_H_^MIM^ exhibited H_2_ production activities of 0.71 ± 0.11 mol H_2_ × mol photosensitizer^−1^ × h^−1^ and 1.1 ± 0.2 mol H_2_ × mol photosensitizer^−1^ × h^−1^, respectively.

## 4 Discussion

Artificial [FeFe]‐hydrogenases of a much smaller size than their natural counterparts might be beneficial for applications because they might be easier to produce or to be plugged into additional circuits, such as those based on DNA technologies.^[^ ^87^, ^88^ ^]^ In addition, characterizing their catalytic features may help to understand the natural enzymes, for example by studying the impacts of the second and higher‐order coordination spheres.^[^ ^25^, ^89^ ^]^ Here, we screened various plant‐type ferredoxins, in which the [2Fe‐2S] cofactor is surrounded by a loop region that separates it from the solvent, potentially providing a natural pocket for the insertion of artificial cofactors. Additionally, natural ferredoxins often interact with various redox partners in cells, suggesting that artificial hydrogenases based on ferredoxin scaffolds might be coupled to other enzymes.

Notably, *Cv*FdxA allowed the cofactor mimic to generate H_2_ at comparably high rates (Figure 2). After introducing its double‐glycine motif (CRGGVC) (Figure 3) into the *Cr*PetF protein, this variant, too, showed H_2_ production activity after incubation with the 2Fe_H_^MIM^ cofactor, and so did three additional ferredoxins that contain the “GGV motif”, either naturally (ferredoxins from *M. conductrix and C. ohadii*) or introduced by genetic modification (*C. anuum* ferredoxin). The observation that only a few of the tested ferredoxins and ferredoxin variants could be combined with the 2Fe_H_^MIM^ complex in a way that allowed H_2_ evolution suggested that these proteins had specific features that provided a suitable environment.

UV‐Vis spectra of the ferredoxins analyzed here revealed that all proteins that allowed 2Fe_H_^MIM^ activation were purified from the *E. coli* host in their apo (*Cv*FdxA; *Co*Fd; *Ca*Fd_GGV_) or mostly apo form (*Cr*PetF_GGV_, *Mc*Fdtr) (Figure 6). In addition to the lack of typical absorption maxima of oxidized [2Fe‐2S] ferredoxins, the spectra of these latter polypeptides showed maxima in the UV region at lower wavelengths than typical ferredoxins. A blue‐shift of the UV absorption of proteins can be the result of changed environments of Tyr, Trp and, to a lesser extent, Phe residues^[^ ^90^ ^]^ and has, for example, been observed upon unfolding of a ferredoxin.^[^ ^91^ ^]^ The UV‐Vis spectra thus suggest that the ferredoxins that developed H_2_ evolution capabilities in the presence of 2Fe_H_^MIM^ had an empty active site pocket and perhaps an altogether different structure than holo‐ferredoxins. Comparing wild‐type *Cr*PetF, *Cv*FdxA, and the variant *Cr*PetF_GGV_, our results agree with the interpretation that the “GGV motif” in the loop that covers the [2Fe‐2S] cluster in plant‐type ferredoxins may play a role in allowing a ferredoxin to render 2Fe_H_^MIM^ catalytically active. Indeed, from all ferredoxin proteins tested here, only those that contained this motif – either naturally or introduced by us – resulted in H_2_‐producing forms after being incubated with the diiron site mimic. This was particularly notable in the case of *Cr*PetF which was able to endow 2Fe_H_^MIM^ with activity only in its variant form *Cr*PetF_GGV_ (Figure 2). We assume that, depending on the protein environment, the flexibility provided by the two consecutive Gly residues may prevent the formation of a stable [2Fe‐2S] cluster in the *E. coli* host. In the case of a minimal peptide maquette based on the loop region of bacterial ferredoxins (CIACGAC), exchanging amino acids that do not coordinate the [4Fe‐4S] cluster that can be assembled on this peptide can lead to low cluster occupancies.^[^ ^92^ ^]^ Although not directly comparable to full‐size ferredoxins, these observations show that the sequence environment of the coordinating Cys residues plays an important role in cluster stabilization. The lack of the natural cluster, in turn, might be the prerequisite for a ferredoxin polypeptide to be able to confine and/or shield 2Fe_H_^MIM^ in a way that allows it to become active, perhaps by incorporating it in the empty active site niche. However, additional structural properties besides the “GGV motif” must play a role because not all ferredoxin variants with this sequence pattern were purified without a [2Fe‐2S] cluster and generated H_2_ in their 2Fe_H_^MIM^‐loaded forms.

To gain insights into the way the diiron site mimic interacts with the ferredoxin proteins, we analyzed the 2Fe_H_^MIM^‐loaded *M. conductrix* ferredoxin (*Mc*Fdtr‐2Fe_H_^MIM^) by FTIR spectroscopy, which revealed the specific CO/CN^−^ signals of the 2Fe_H_^MIM^ complex. Spectra of *Mc*Fdtr‐2Fe_H_^MIM^ did not show the typical band patterns observed for the H‐cluster in [FeFe]‐hydrogenases (Figure S4B, Supporting Information). However, when we compared its spectrum with that of the free 2Fe_H_^MIM^ complex, we noted that the presence of the ferredoxin protein resulted in subtle, yet specific band shifts (Figure 5A). This specifically shifted band pattern could be recapitulated when the 2Fe_H_^MIM^ complex was either dried on the ATR FTIR crystal (Figure S1A,B, Supporting Information) or subjected to an aqueous DMSO solution (Figure 5B), suggesting that it resulted from a reduced solvation of the 2Fe_H_^MIM^ compound and a more hydrophobic environment. The similarities of these latter spectra of 2Fe_H_^MIM^ to those of *Mc*Fdtr‐2Fe_H_^MIM^, and the observation that the band pattern of *Mc*Fdtr‐2Fe_H_^MIM^ did not change upon drying (Figure S1D, Supporting Information) support the assumption that the *Mc*Fdtr protein shields the mimic from the solvent.

Both *Mc*Fdtr‐2Fe_H_^MIM^ and *Cr*PetF_GGV_‐2Fe_H_^MIM^ revealed much higher residual H_2_ evolution activities after exposure to air than the naturally very O_2_‐sensitive [FeFe]‐hydrogenase *Cr*HydA1 (Figure 4). This observation, too, suggests that the diiron site mimic interacts with the ferredoxin scaffolds, as free 2Fe_H_^MIM^ is also O_2_‐sensitive and degrades within 30 to 60 min in aqueous solution.^[^ ^17^ ^]^ The protein fold may provide protection from O_2_ accessing the cofactor. Alternatively, it is reasonable to suggest that the transport of protons to 2Fe_H_^MIM^ is different in the non‐native protein environments. Both for free diiron site analogs and for [FeFe]‐hydrogenases, protonation was shown to be one important aspect of the O_2_‐induced inactivation process.^[^ ^16^, ^17^, ^93^ ^]^

**Figure 7:**
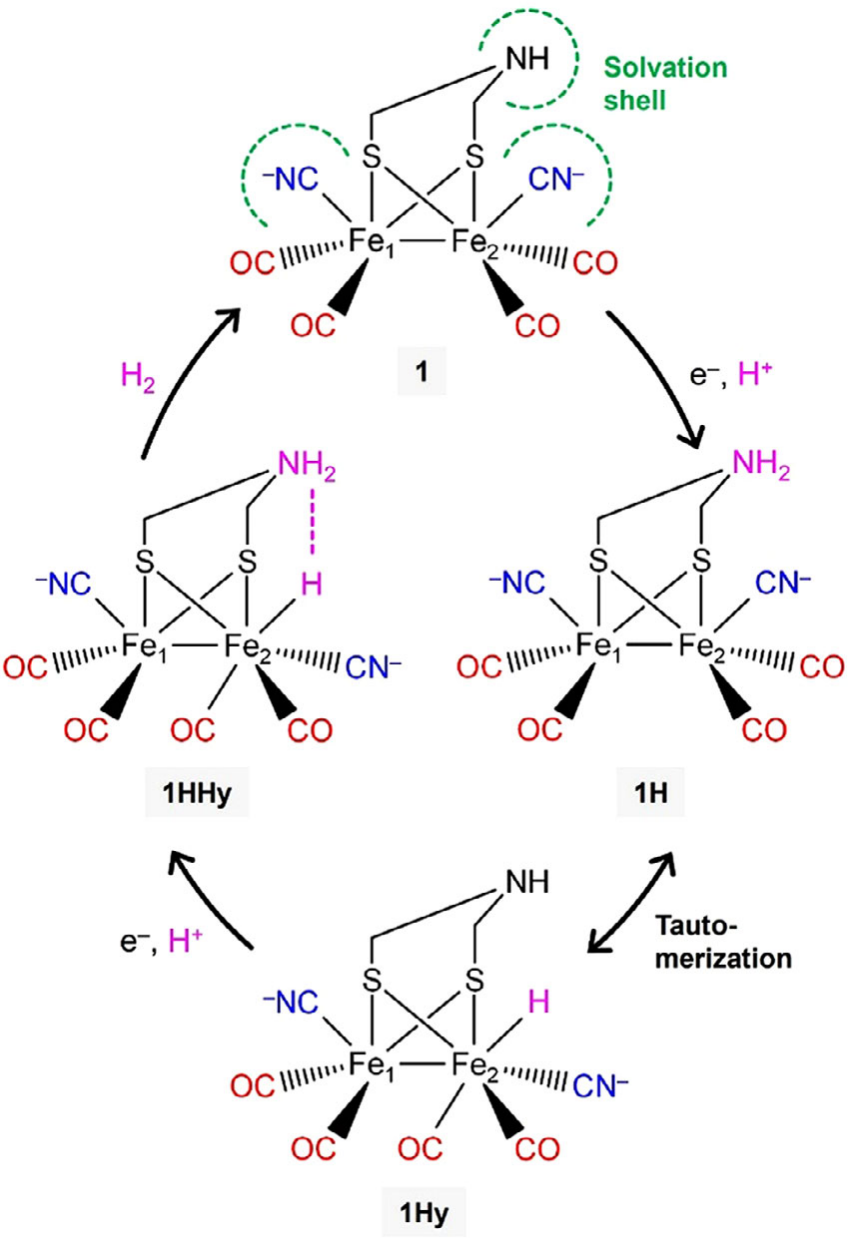
Proposed mechanism of H_2_ evolution by saturated azadithiolate diiron complexes. Studies that employed acids of different p*K*_a_ values combined with electrochemical reduction suggested the mechanism shown here.^[^ ^94^ ^]^ The figure depicts the differently protonated and reduced forms of 2Fe_H_^MIM^, showing the protons in pink letters. **1**: Most likely rotational isomer of 2Fe_H_^MIM^ in aqueous solution. Water will hydrogen‐bond with the ADT and CN^−^ ligands and form a solvation shell (green). **1H**: ADT‐protonated; **1Hy**: hydride‐binding; **1HHy**: protonated and hydride‐binding containing the Fe‐H/NH_2_ frustrated Lewis pair (dashed line). Protic solvents like H_2_O may influence the equilibrium between **1H** and **1Hy** (tautomerization).

For the time being, a robust hypothesis on how the ferredoxin scaffolds allowed 2Fe_H_^MIM^ to develop proton‐reducing activity cannot be put forward, because structural information is required that could give hints to a possible proton transfer pathway. Our efforts to obtain crystal structures of *Mc*Fdtr‐2Fe_H_^MIM^ and *Cr*PetF_GGV_‐2Fe_H_^MIM^ have been unsuccessful to date. Notably, the FTIR spectra of *Mc*Fdtr–2Fe_H_^MIM^ did not reveal a signal for a *µ*CO ligand, which is usually observed between 1860 and 1790 cm^−1^.^[^ ^82^, ^83^, ^84^ ^]^ Maturating apo [FeFe]‐hydrogenases with the cofactor analog results in the release of one of the four CO ligands of the mimic and the formation of *µ*CO.^[^ ^33^ ^]^ We could only detect minor amounts of CO being released upon incubating *Mc*Fdtr with 2Fe_H_^MIM^ (Figure S5, Supporting Information), which is in agreement with the subtle differences in the IR spectra. Clearly, 2Fe_H_^MIM^ stayed in its fully saturated form with four CO ligands and two CN^−^ ligands. In solution, ADT‐type diiron complexes have been shown to catalyze proton reduction at high overpotentials^[^ ^94^ ^]^ or in the presence of very strong reductants.^[^ ^59^ ^]^ The proposed mechanism includes protonation of the ADT ligand and formation of a bridging hydride (*µ*H) or terminal hydride (tH), both of which recombine to H_2_ eventually (**Figure** **7**).^[^ ^94^, ^95^ ^]^ It is not entirely surprising to measure H_2_ evolution with *Mc*Fdtr‐2Fe_H_^MIM^, despite the unchanged IR spectra discussed above. However, we were surprised to find catalytic activity at mild conditions. Optimized proton transfer toward 2Fe_H_^MIM^ within *Mc*Fdtr may facilitate catalysis at less negative reduction potentials, which certainly plays a role in hydrogenases,^[^ ^96^ ^]^ and the lack of activity in the PDT‐loaded ferredoxins emphasizes the role of the ADT ligand in proton shuttling. Moreover, we speculate that the rotational freedom of ligands affects the probability of hydride formation, a concept that was introduced for [FeFe]‐hydrogenases by Fourmond et al. in 2014.^[^ ^97^ ^]^ Using the example of the tH‐type mechanism,^[^ ^94^ ^]^ Figure 7 illustrates that this would relate to the tautomerization step between the ADT‐protonated (**1H**) and the hydride‐binding 2Fe_H_^MIM^ complex (**1Hy**). In an aqueous solution, the CN^−^ ligands of 2Fe_H_^MIM^ interact with solvent molecules and stabilize rotational isomer **1** (“solvation shell” in Figure 7), which is in agreement with our DFT calculations (Figure S2, Supporting Information) and the crystal structure of 2Fe_H_^MIM^.^[^ ^9^ ^]^ In a solvent‐protected environment like the *Mc*Fdtr active site, however, this stabilization is missing, and increased ligand rotation could increase the chance of hydride binding – the complex “samples” more conformations per time – which would explain the high activity of the ferredoxin hybrid. Additionally, the lack of solvent molecules will strengthen the Fe‐H/NH_2_ frustrated Lewis pair of the double‐protonated 2Fe_H_^MIM^ complex **1HHy** (Figure 7), similar to the situation in the [FeFe]‐hydrogenase active site.^[^ ^98^ ^]^ It is notable that 2Fe_H_^MIM^ did not show activity when loaded onto the maturase protein HydF, whereas the respective PDT and propanediselenol complexes did result in activity when combined with HydF,^[^ ^36^, ^37^, ^79^ ^]^ despite lacking a protonable ADT ligand. These data emphasize the importance of the protein fold. In the future, this comparison may give clues on the mechanisms that enable the diiron site analogs to be activated in the different protein hosts.

Employing ferredoxins as hosts for 2Fe_H_^MIM^ appears promising in that some of the hybrid proteins reached quite high activities when compared with similar systems (Table S2, Supporting Information). Designing the proteins in a way that allows an even higher activity, for example by introducing a proton transfer pathway, would be the next step. A sustainable electron delivery system is another important aspect of any redox enzyme.^[^ ^99^ ^]^ Here, we tested whether *Mc*Fdtr‐2Fe_H_^MIM^ and *Cr*PetF_GGV_‐2Fe_H_^MIM^ are able to receive electrons from PSI, which is a natural electron donor of plant‐type ferredoxins, or the chemical photosensitizer proflavine, which has been used as a “PSI mimic” in hydrogenase research before.^[^ ^57^, ^100^ ^]^ Indeed, both ferredoxin‐2Fe_H_^MIM^ hybrids did evolve H_2_ photocatalytically in these systems, albeit with lower rates than when the natural electron transport chain was simulated by using wild‐type *Cr*PetF and *Cr*HydA1. Our results suggest that 2Fe_H_^MIM^ binds to the ferredoxins instead of their [2Fe‐2S] clusters, which likely affects the redox potential as discussed above. This mismatch in electric potentials could explain the inferior reduction efficiency with PSI or proflavine. However, our results show that the ferredoxin‐2Fe_H_^MIM^ complexes still interact with PSI, suggesting that their structure does not deviate too much from the natural PetF structure. The observation that *Mc*Fdtr‐2Fe_H_^MIM^, although having a higher activity when tested with the NaDT‐ and MV‐based assay, was less active when coupled to PSI indicates that either the natural protein or the shortened recombinant protein employed here is less suited to interact with PSI efficiently.

## 5 Conclusion

We have shown that naturally occurring plant‐type ferredoxins can endow a chemical analog of the active site of [FeFe]‐hydrogenases, 2Fe_H_^MIM^, with a comparably high catalytic capacity to generate H_2_. According to photometric analyses, the lack of the natural [2Fe‐2S] cluster of the ferredoxins is a prerequisite for this to happen, and FTIR data indicate that the active site mimic is shielded from the solvent. This suggests that the apo‐ferredoxins either integrated the chemical analog into their empty active site pocket or featured unnatural structures that formed a suitable environment for 2Fe_H_^MIM^. In contrast to the diiron site of the natural H‐cluster of [FeFe]‐hydrogenases, the 2Fe_H_^MIM^ complex stayed in its ligand‐saturated form. This poses questions regarding the reaction mechanism that results in the H_2_ evolution of the ferredoxin‐2Fe_H_^MIM^ hybrids. Our current hypothesis is that the lack of solvent molecules within the ferredoxin polypeptide promotes ligand rotation and the formation of a terminal hydride intermediate. Gaining structural information on how exactly the mimic binds to the ferredoxin polypeptides will be an important next step and will help to elucidate the functional principles of the hybrid proteins in comparison to natural [FeFe]‐hydrogenases. This, in turn, would assist the rational design of optimized ferredoxin scaffolds. Our observation that the ferredoxin‐2Fe_H_^MIM^ proteins were still able to receive electrons from PSI indicates that these proteins might be integrated into modular electron delivery systems for reductive processes, as has been shown for other natural PSI acceptor proteins equipped with H_2_ evolving catalysts before. A distinguishing feature of the system we established here is the reliance on the almost natural diiron site which can be generated sustainably through enzymatic synthesis. Whether the enzymatic maturation system could transfer the diiron site to one of our ferredoxin scaffolds remains to be tested. If this were the case, chemical syntheses could be avoided and the footprint of artificial H_2_ production systems could be greatly reduced.

## Conflict of Interest

The authors declare no conflict of interest.

## Supporting information



Supporting Information

## Data Availability

The data that support the findings of this study are available from the corresponding author upon reasonable request.
