# Supplementary material for: Hydrogen‐Producing Catalysts Based on Ferredoxin Scaffolds
Source: Adv Sci (Weinh). 2025 Jun 17;12(33):e01897. doi: 10.1002/advs.202501897 (PMC12412533; doi:10.1002/advs.202501897)
Supplement: Supplementary file 1 — Supporting Information [file ADVS-12-e01897-s001.pdf]

ADVANCED SCIENCE
Open Access

Supporting Information

for *Adv. Sci.*, DOI 10.1002/advs.202501897

Hydrogen-Producing Catalysts Based on Ferredoxin Scaffolds

Yiting She, Vera Engelbrecht, Jacek Kozuch, Ulf-Peter Apfel, Sven T. Stripp, Anja Hemschemeier and Thomas Happe**

Supporting Information

for

Hydrogen Producing Catalysts Based on Ferredoxin Scaffolds

by

Yiting She, Vera Engelbrecht, Jacek Kozuch, Ulf-Peter Apfel, Sven T. Stripp, Anja Hemschemeier, Thomas Happe

Content:

Table S1. Codon-optimized sequences for recombinant ferredoxin production.
Table S2. H_2 evolution turnover frequencies (TOFs) of several artificial hydrogenases based on natural protein hosts.
Table S3. Measured and DFT-calculated frequencies of the CN^- and CO ligands.
Table S4. Assignment of normal modes of $2Fe_H^{MIM}$ isomer 1aa to CN^- and CO ligands.
Figure S1. FTIR spectra of $2Fe_H^{MIM}$ and *Mc*Fdtr-$2Fe_H^{MIM}$ upon dehydration.
Figure S2. Isomers of $2Fe_H^{MIM}$ and DFT-calculated spectra.
Figure S3. Full FTIR spectra of *Mc*Fdtr-$2Fe_H^{MIM}$, *Mc*Fdtr and $2Fe_H^{MIM}$.
Figure S4. FTIR spectra of DMSO and of *Cr*HydA1.
Figure S5. CO release upon mixing *Mc*Fdtr with $2Fe_H^{MIM}$.
References

Table S1. Codon-optimized sequences employed to recombinantly produce the ferredoxins used in this study.
The regions colored in yellow represent the Strep-tag-encoding parts. The linker coding for the factor Xa cleavage site is underlined. In the case of *Cv*FdxA, the Ala-Ser linker-encoding codons are written in italics. Abbreviations used are the same as in Table 1 in the main text.

Name	Coding sequences
***Cv*FdxA**	ATGCAGGTTACCTTTGTTAGCGAAGAAACCGGTATGAGCAAAACCGCAGAAATTGGTCCGAATGAATATCTG<br>CTGGATGGTGCAGATGAACATCGTATTGAAGTTAATGCAAGCTGTCGTGGTGGTGTTTGTGGCACCTGTGTT<br>AGCAAACTGCGTAGCGGTCAGGTTGATCTGGAATGGCTGGATGTTCTGGATGAAGGTAGCGTTCTGAGCCA<br>AGAACAGGATGGTTATATTCTGCCGTGTAGCGCCAAACCGCTGAGCGATTGTGTTGTTGAAATTAAA*GCAAG*<br>*T*TGGAGCCATCCGCAGTTTGAGAAATAA
***Mc*Fdtr**	ATGGCAAGCTGGAGCCATCCGCAGTTCGAAAAAATTGAAGGTCGTTATAAAGTGACCTTCGTGGATCAGAC<br>CAGTGGCGCCGAACGTACCTGTGAAATTAGCGCAGATGATTATCTGCTGGATGGCGCAGATGCAAGCCGCA<br>TTGATCTGAATGCAAGTTGTCGTGGCGGTGTGTGTGGTACCTGCGTGAGTAAACTGCGCAGTGGCCAGGTT<br>GATATGGAATGGCTGAGTGTGCTGGATGATGGTAATGTGCTGAGTCAGGAACAGGTGGCAGCAGGTTATAT<br>TCTGCCGTGCAGTGCAAAACCGCTGAGTGATTGTGTTGTTGAAACCAGCAAAGATTGGGGCCATACCTAA
***Co*Fd**	ATGGCAAGTTGGAGCCATCCGCAGTTCGAAAAAATTGAAGGTCGTTATAAAGTGACCTTCATTCGCGATGGT<br>AAAGAAAAAACCTGCGAAGTTGGTCCGAATGATTATATGCTGGATGCCGCCGATGCCAGTCGTATTGAACTG<br>AATGCAAGTTGCCGTGGTGGTGTGTGTGGCACCTGTGTGAGCAAACTGGTGAGTGGTACCGTTGATAATGA<br>ATGGCTGGAAGTTCTGGATGATGGTAATGTGCTGAGTAAAGAACAGATTGCCGCAGGTTATATTCTGCCGTG<br>CAGCGCAAAACCGACCAGTGATTGTGTGGTTGAAGCCAATAGTGATTGGGGCGTGCATACCATTGAAAAAT<br>GGAGCCATACCGCGCGCGCGTAA
***Ca*Fd$_{GGV}$**	ATGGCCAGCTGGAGCCATCCGCAGTTCGAAAAAATTGAAGGCCGCTATAAAGTGAAACTGATTACCCCGGA<br>TGGTCCGATTGAATTCGATTGTCCGGATAATGTGTATATTCTGGATCAGGCAGAAGAAGCAGGTCATGATCT<br>GCCGTATAGCTGCCGTGGTGGTGTGTGCAGCAGCTGTGCCGGTAAAATTGCAGGCGGCGCCGTTGATCAG<br>ACCGATGGCAACTTCCTGGATGATGATCAGCTGGAAGAAGGTTGGGTTCTGACCTGCGTGGCCTATCCGCA<br>GAGTGATGTGACCATTGAAACACATAAAGAAGCCGAACTGGTGGGTTAA
***Cm*Fd$_{GGV}$**	ATGGCAAGTTGGAGCCATCCGCAGTTCGAAAAAATTGAAGGCCGTTATAAAATCCAGCTGGTTAATCAGAAA<br>GAAGGCATTGATGTTACCATTCAGTGTGCAGGTGATCAGTATATTCTGGATGCAGCAGAAGAACAGGGTGTT<br>GATCTGCCGTATAGCTGTCGTGGCGGCGTGTGTAGCACCTGCGCCGGTAAACTGGTTAAAGGTAGCGTGG<br>ATCAGAGTGATCAGAGCTTCCTGGATGAAGATCAGATTAGCAAAGGCTTCATTCTGACCTGTGTTGCCTATC<br>CGACCAGTGATTGCGTTATTCAGACCCATCAGGAAGAAGCCCTGTATTAA
***Ap*Fd$_{GGV}$**	ATGGCAAGTTGGAGCCATCCGCAGTTCGAAAAAATTGAAGGTCGTTATAAAGTGACCCTGATTAATGAAGCC<br>GAAGGTATTAATGAAACCATTGATTGTGATGATGACACCTATATTCTGGATGCCGCAGAAGAAGCAGGTCTG<br>GATCTGCCGTATAGTTGTCGTGGCGGCGTGTGTAGTACCTGTGCCGGTACCATTACCAGTGGCACCATTGA<br>TCAGAGCGATCAGAGCTTCCTGGATGATGATCAGATTGAAGCAGGTTATGTGCTGACCTGCGTGGCCTATC<br>CGACCAGCGATTGCACCATTAAAACACATCAGGAAGAAGGTCTGTATTAA
***Mm*Fd$_{GGV}$**	ATGGCAAGTTGGAGTCATCCGCAGTTCGAAAAAATTGAAGGCCGTTATAAAGTGACCCTGAAAACACCTAGC<br>GGTGATAAAGTTATTGAATGTGCAGATGATGTGTATATTCTGGATGCAGCCGAAGAAGCAGGCCTGGATCTG<br>CCGTATAGTTGCCGCGGTGGTGTGTGTAGCAGTTGCGCAGGTAAAGTGGAAGCCGGCACCATTGATCAGA<br>GCGATCAGAGCTTCCTGGATGATAGTCAGATGGGCAATGGCTTCGTGCTGACCTGTGTGGCCTATCCGACC<br>AGTGATTGTACCATTAGTACCCATCAGGAAGAAGCCCTGTATTAA

Table S2. H_2 evolution turnover frequencies (TOFs) of several artificial hydrogenases based on natural protein hosts.

The table lists examples of mostly unmodified natural proteins serving as hosts for H_2 generating catalysts. The interested reader is referred to review articles that list additional chemical or semi-biological artificial H_2 producing catalysts.[1-3]. Abbreviations: pdt: propanedithiolate; pdSe: propane di-selenol; $[Ru(bpy)_3]^{2+}$: Tris(bipyridine)ruthenium, a photosensitizer. The indicated TOFs are those reported in the cited studies unless indicated otherwise.

Protein	Cofactor	TOF [mol H_2 × mol catalyst^{-1} × min^{-1}]	Buffer conditions and electron sources	Reference
**Cytochrome *c***	$[(\mu\text{-S-Cys})_2Fe_2(CO)_6]$	2.1 (calculated from the initial rate)	0.05 M Tris/HCl at pH 4.7; $[Ru(bpy)_3]^{2+}$ and ascorbate (light)	[4]
**Nitrobindin**	maleimide-functionalized $[(\mu\text{-S})_2Fe_2(CO)_6]$	2.3 (calculated from the initial rate)	0.05 M Tris/HCl, pH 4.0 (aq); $[Ru(bpy)_3]^{2+}$ and ascorbate (light)	[5]
***Spinacia oleracea* ferredoxin**	cobaloxime	1 (per $[Ru(bpy)_3]^{2+}$), or 0.43 (per ferredoxin)[(a)]	0.01 M MES pH 6.3, $[Ru(bpy)_3]^{2+}$, covalently bound to the ferredoxin, and ascorbate (light)	[6]
***Synechococcus lividus* apo-flavodoxin**	cobaloxime	0.5 (per $[Ru(bpy)_3]^{2+}$)[(a)]	0.01 M MES pH 6.3, $[Ru(bpy)_3]^{2+}$, covalently bound to the flavodoxin, and ascorbate (light)	[7]
**HydF from *Thermotoga maritima***	$[Fe_2(pdt)(CO)_4(CN)_2]^{2-}$	0.5 – 1 (initial rate)	0.1 M potassium phosphate buffer, pH 6 (aq), dithionite and methyl viologen	[8]
**HydF from *Thermosipho melanesiensis***	$[Fe_2(pdt)(CO)_4(CN)_2]^{2-}$	0.3 (initial rate)	0.1 M potassium phosphate buffer, pH 6 (aq); dithionite and methyl viologen	[9]
**HydF from *T. melanesiensis***	$[Fe_2(pdSe)(CO)_4(CN)_2]^{2-}$	0.65 (calculated from the first 5 min of the assay)	0.1 M phosphate buffer, pH 6 (aq); dithionite and methyl viologen	[10]

[(a)]calculated here from the original rates indicated in h^{-1}

Table S3. Measured and DFT-calculated frequencies of the CN^{-} and CO ligands (in cm^{-1}).
See Figure S2 for more information on the proceeding and the corresponding calculated spectra. Isomer 3ae did not converge in the polarizable continuum model (PCM) and was therefore not considered further (gray letters).

exp.	*vacuum*					*PCM*				
	**1**aa	**2**ea	**3**ae	**4**ee^{cis}	**5**ee^{trans}	**1**aa	**2**ea	**3**ae	**4**ee^{cis}	**5**ee^{trans}
2058<br>2038	2058	2079<br>2054	2081<br>2058	2083	2080	2058<br>2052	2066<br>2052	2067<br>2055	2068<br>2063	2067<br>2062
1984<br>1952<br>1918	1926<br>1892<br>1866	1922<br>1890<br>1866<br>1840	1921<br>1892<br>1862<br>1836	1914<br>1885<br>1842<br>1835	1914<br>1894<br>1837<br>1833	1917<br>1872<br>1842<br>(1839)	1911<br>1872<br>1843<br>1831	1909<br>1862<br>1839<br>1818	1904<br>1868<br>1830<br>(1826)	1869<br>1827<br>(1825)

Table S4. Assignment of normal modes of $2Fe_H^{MIM}$ isomer 1aa to CN^{-} and CO ligands.
Normal modes of $2Fe_H^{MIM}$ isomer **1**aa were assigned to CN^{-} and CO ligands based on the relative amplitudes of mass-weighted cartesian displacements. Normal modes were localized to >95% on the CO/CN^{-} ligands (fractions below 5% are not shown). Negative signs indicate anti-symmetric coupling with respect to CO/CN^{-} stretches with positive percentage. See Figure S2A for $2Fe_H^{MIM}$ isomer **1**aa, whose CO/CN^{-} ligands are numbered from 1 to 6. **2058 cm^{-1}:** symmetric coupling of both CN^{-} stretches, mostly localized on the left CN^{-} ligand (3); **2052 cm^{-1}:** anti-symmetric coupling of both CN^{-} stretches, mostly localized on the right CN^{-} ligand (4); **1917 cm^{-1}:** collective symmetric coupling of all CO stretches; **1872 cm^{-1}:** collective anti-symmetric coupling of all CO stretches (the COs on each Fe ion are symmetric; **1842 cm^{-1}** and **1839 cm^{-1}**: anti-symmetric CO stretches on the right (1 and 2) and left Fe ion (5 and 6), respectively.

ν		*left Fe ion*			*right Fe ion*		
**exp.**	**cal.**	**CO**	**CO**	**CN**	**CN**	**CO**	**CO**
2058	2058	-	-	82%	10%	-	-
2038	2052	-	-	11%	-83%	-	-
1984	1917	24%	21%	-	-	26%	24%
1952	1872	30%	22%	-	-	-27%	-20%
1918	1842	-	-	-	-	45%	-54%
	1839	44%	-56%	-	-	-	-
**number:**		1	2	3	4	5	6

Figure S1. FTIR spectra of $2Fe_H^{MIM}$ and *Mc*Fdtr-$2Fe_H^{MIM}$ upon dehydration.

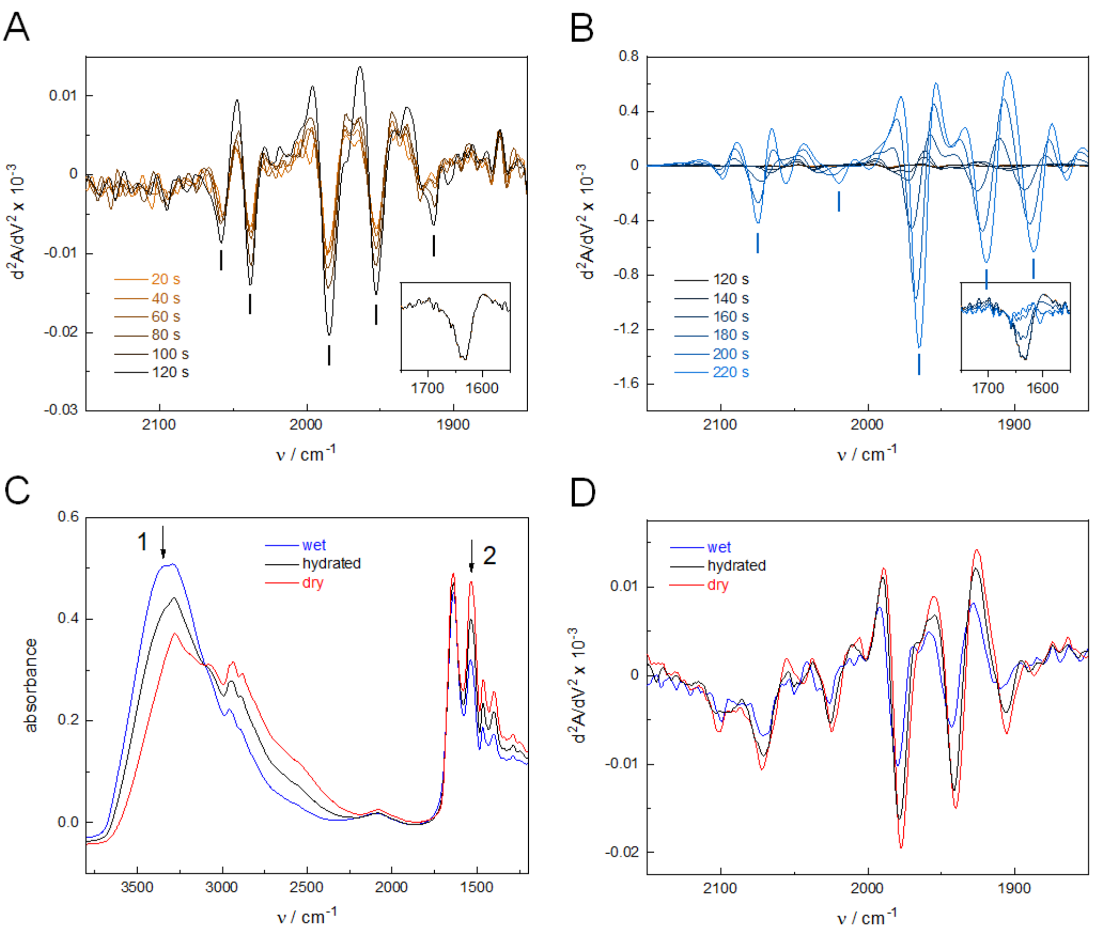

Figure S1. Second derivative ATR FTIR spectra of the $2Fe_H^{MIM}$ complex and of *Mc*Fdtr-$2Fe_H^{MIM}$ upon dehydration. The free $2Fe_H^{MIM}$ complex (100 g × L^{-1} in water) was dried on the ATR FTIR crystal by purging it with a N_2 gas stream (1 L × min^{-1}). Spectra were recorded every 20 seconds. **A** shows the early stages of drying, **B** the subsequent drying process. Note that the H_2O bending mode at 1640 cm^{-1} (insets in **A** and **B**) was apparently unchanging in the early stages of drying but decreased rapidly in the later stages. The loss of solvent is associated with significant spectral changes, clearly visible in **B**. **C** and **D**: Following established protocols,[11] *Mc*Fdtr-$2Fe_H^{MIM}$ solution (650 µM in 0.1 M Tris-HCl, pH 8.0) was dried and then rehydrated to a protein film before different hydration levels were adjusted. 'Dry' refers to a protein film under pure N_2, 'wet' to the same protein film exposed to a N_2 gas stream that was routed through a wash bottle containing water, and 'hydrated' depicts an intermediate state purged with N_2 gas mixed with N_2 aerosol at an approximate 1 : 1 ratio of dry and wet gas. The humidity of the gas phase in contact with the protein film is reflected by the stronger water signal (arrow 1, 3350 cm^{-1}) or a smaller amide II band (arrow 2, 1540 cm^{-1}) indicated in **C**. The intensity of the cofactor bands in **D** followed the trend of the amide II band in **C**, however, the frequency shifts of the isolated complex observed under similar conditions (**B**) were not observed for *Mc*Fdtr-$2Fe_H^{MIM}$ (**D**).

Figure S2. Isomers of $2Fe_H^{MIM}$ and DFT-calculated spectra.

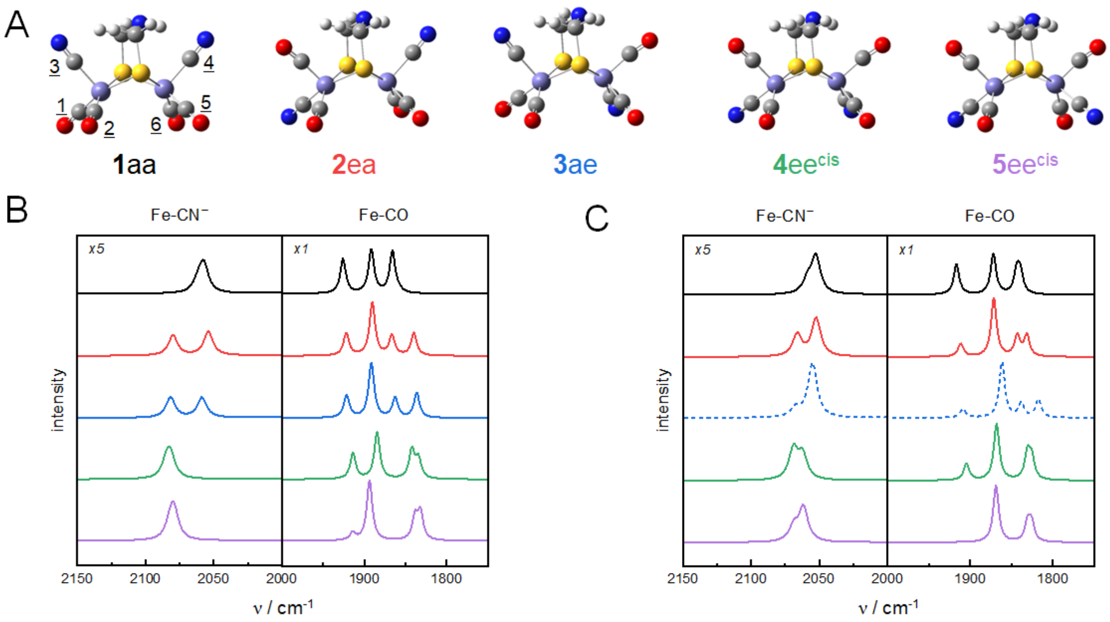

Figure S2. Calculation and assignment of normal modes. (A) We considered five rotational isomers of $2Fe_H^{MIM}$ and calculated the CN^- and CO normal modes in either vacuum or water (polarizable continuum model, PCM). Isomers differ with respect to the position of the CN^- ligands: apical-apical (**1**aa), equatorial-apical (**2**ea), apical-equatorial (**3**ae), or both CN^- ligands in equatorial position, either in *cis* or *trans* configuration (**4**ee^{cis} or **5**ee^{trans}, respectively). The graphs below depict the calculated CN^- and CO spectra of isomers 1 to 5 in vacuum **(B)** and with the PCM **(C)**. The CN^- regime is magnified by a factor of five for ease of comparison. See Material & Methods for computational details and Table S3 for a list of computed frequencies. The experimental spectrum of $2Fe_H^{MIM}$ is characterized by two bands in the CN^- and three bands in the CO regime (Figure 5, main text). Among the gas-phase spectra **(B)**, only **2**ea and **3**ae show individual contributions from the CN^- ligands; however, **1**aa is the only isomer with three CO bands. Therefore, none of these patterns fit the experimental data directly. Calculating the solvated complexes **(C)** resulted in discernible, albeit partially overlapping CN^- bands for all isomers. However, isomer **2**ea shows four CO bands and isomer **5**ee^{trans} shows only two CO bands, and the lower energy band consists of two overlapping contributions. These calculations disagree with the experimental data. Note that isomer **3**ae (dashed blue line) did not converge in the PCM and adopted a 'twisted' structure, which will not be considered further. Only isomers **1**aa and **4**ee^{cis} reproduced fitting IR spectra including two CN^- and three CO bands, from which the lowest energy band consists of two overlapping contributions. Structural analysis by small molecule X-ray crystallography of $[Fe_2[(SCH_2)_2NH](CN)_2(CO)_4]$ – which we refer to as $2Fe_H^{MIM}$ here – led to an electron density distribution best compatible with the apical-apical configuration.[12] Therefore, we assign the normal modes according to **1**aa as described in Table S4.

Figure S3. FTIR spectra of *Mc*Fdtr-$2Fe_H^{MIM}$, *Mc*Fdtr, and $2Fe_H^{MIM}$.

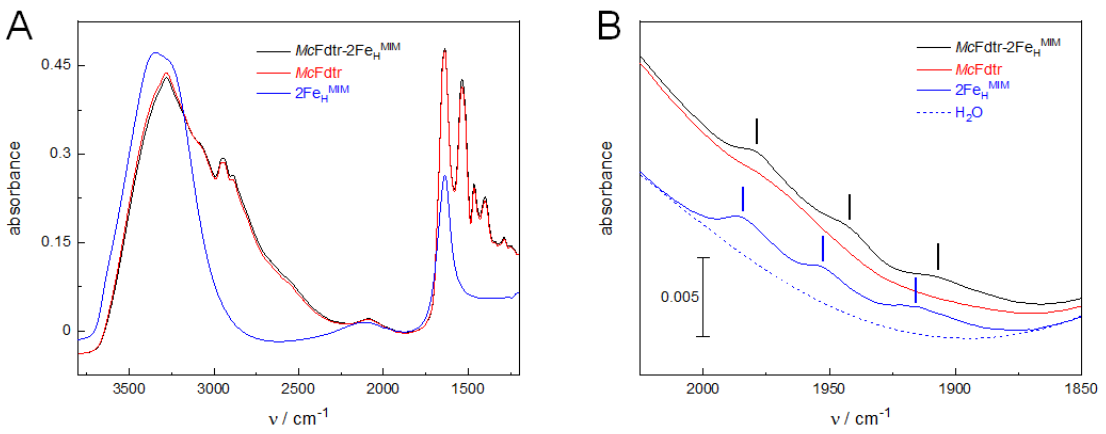

Figure S3. FTIR spectra of *Mc*Fdtr-$2Fe_H^{MIM}$, *Mc*Fdtr, and $2Fe_H^{MIM}$. (A) Comparison of ATR FTIR spectra for $2Fe_H^{MIM}$ in diluted, aqueous solution (10 g × L^{-1}, blue trace) and hydrated protein films of *Mc*Fdtr and *Mc*Fdtr-$2Fe_H^{MIM}$ (red and black traces, respectively). All data show a similar water content (around 3350 cm^{-1}), which results in similar protein concentrations for the ferredoxin films (amide II at 1540 cm^{-1}). **(B)** Close-up in the frequency regime of the CO ligands. The bands appear with a similar intensity in the $2Fe_H^{MIM}$ and *Mc*Fdtr-$2Fe_H^{MIM}$ samples. No such signals were observed in *Mc*Fdtr and H_2O.

Figure S4. FTIR spectra of DMSO and $2Fe_H^{MIM}$ (A), and of *Cr*HydA1 and $2Fe_H^{MIM}$ (B)

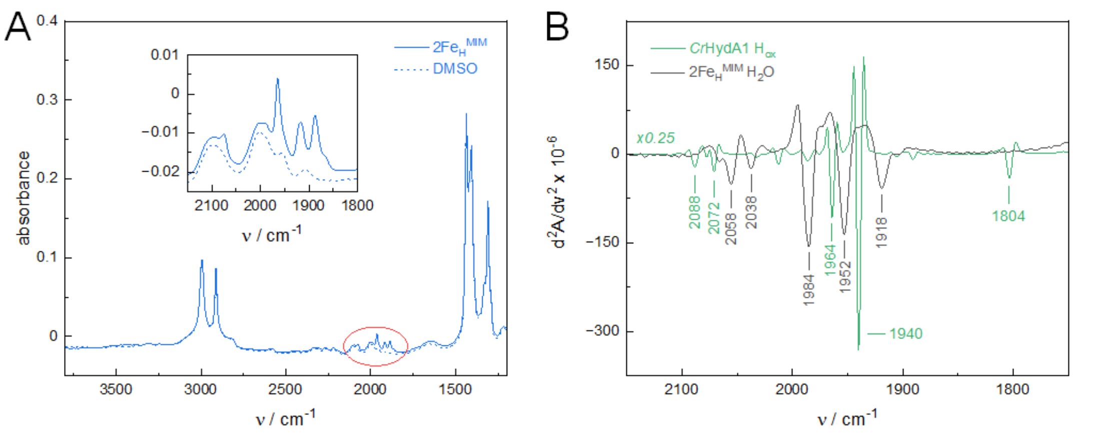

Figure S4. FTIR spectra of DMSO and $2Fe_H^{MIM}$, and a comparison of FTIR spectra of $2Fe_H^{MIM}$ and *Cr*HydA1. (A) Full FTIR absorbance spectra of DMSO and DMSO-dissolved $2Fe_H^{MIM}$ (100 g × L^{-1}). The inset depicts a close-up in the frequency regime of the CO/CN^- ligands. The solvent does not interfere with the spectrum of $2Fe_H^{MIM}$ by sharp bands. **(B)** Comparison of second-derivative spectra of $2Fe_H^{MIM}$ in water (black trace) and of the [FeFe]-hydrogenase *Cr*HydA1 (green trace) in the frequency regime of the CO/CN^- ligands. The spectra are scaled for comparison. The band positions of *Cr*HydA1 are assigned to the oxidized resting state, H_{ox}, including the marker band of μCO at 1804 cm^{-1}. No such signals are observed for $2Fe_H^{MIM}$.

Figure S5. CO release upon mixing *Mc*Fdtr with 2Fe$_H^{MIM}$.

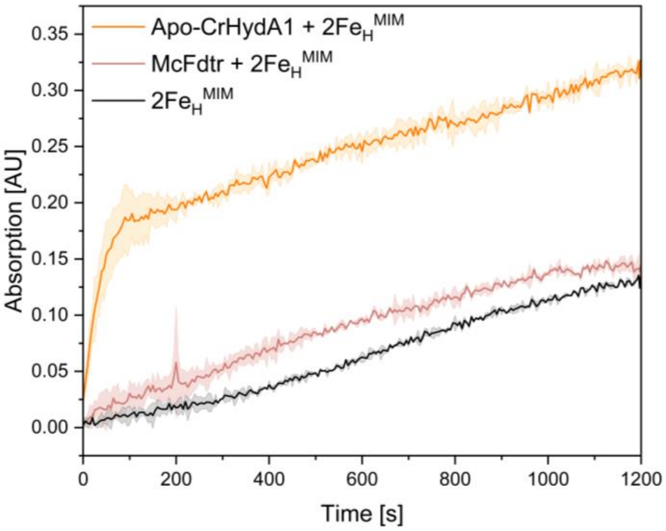

Figure S5. CO-release upon mixing *Mc*Fdtr or 'apo' *Cr*HydA1 with 2Fe$_H^{MIM}$. CO-release tests were conducted by mixing 6 µM *Mc*Fdtr (coral) or 2 µM 'apo' *Cr*HydA1 (i.e., equipped with the [4Fe-4S] subcluster 4Fe$_H$ only; orange) with the 2Fe$_H^{MIM}$ complex (20 µM). CO release was monitored by observing the conversion of deoxyhemoglobin (14 µM) to hemoglobin-CO (Hb-CO), indicated by the increase in absorption at 419 nm for Hb-CO over 1200 s at 22°C. The free 2Fe$_H^{MIM}$ complex (black) served as a control. All molecules were present in 0.1 M potassium phosphate buffer, pH 6.8, supplemented with 2 mM sodium dithionite. Experiments were performed in biological duplicate, and the standard deviation is represented as a shaded area around the lines.

References cited in the supporting information

[1] G. Caserta, S. Roy, M. Atta, V. Artero, M. Fontecave, Artificial hydrogenases: biohybrid and supramolecular systems for catalytic hydrogen production or uptake, *Curr Opin Chem Biol* **2015**, *25*, 36, https://doi.org/10.1016/j.cbpa.2014.12.018.

[2] L. Leone, G. Sgueglia, S. La Gatta, M. Chino, F. Nastri, A. Lombardi, Enzymatic and Bioinspired Systems for Hydrogen Production, *Int J Mol Sci* **2023**, *24* (10), https://doi.org/10.3390/ijms24108605.

[3] F. Schwizer, Y. Okamoto, T. Heinisch, Y. Gu, M. M. Pellizzoni, V. Lebrun, R. Reuter, V. Köhler, J. C. Lewis, T. R. Ward, Artificial Metalloenzymes: Reaction Scope and Optimization Strategies, *Chem Rev* **2018**, *118* (1), 142, https://doi.org/10.1021/acs.chemrev.7b00014.

[4] Y. Sano, A. Onoda, T. Hayashi, A hydrogenase model system based on the sequence of cytochrome c: photochemical hydrogen evolution in aqueous media, *Chem Commun (Camb)* **2011**, *47* (29), 8229, https://doi.org/10.1039/c1cc11157d.

[5] A. Onoda, Y. Kihara, K. Fukumoto, Y. Sano, T. Hayashi, Photoinduced Hydrogen Evolution Catalyzed by a Synthetic Diiron Dithiolate Complex Embedded within a Protein Matrix, *ACS Catal* **2014**, *4* (8), 2645, https://doi.org/10.1021/cs500392e.

[6] S. R. Soltau, J. Niklas, P. D. Dahlberg, O. G. Poluektov, D. M. Tiede, K. L. Mulfort, L. M. Utschig, Aqueous light driven hydrogen production by a Ru-ferredoxin-Co biohybrid, *Chem Commun (Camb)* **2015**, *51* (53), 10628, https://doi.org/10.1039/c5cc03006d.

[7] S. R. Soltau, P. D. Dahlberg, J. Niklas, O. G. Poluektov, K. L. Mulfort, L. M. Utschig, Ru-protein-Co biohybrids designed for solar hydrogen production: understanding electron transfer pathways related to photocatalytic function, *Chem Sci* **2016**, *7* (12), 7068, https://doi.org/10.1039/c6sc03121h.

[8] V. Artero, G. Berggren, M. Atta, G. Caserta, S. Roy, L. Pecqueur, M. Fontecave, From enzyme maturation to synthetic chemistry: the case of hydrogenases, *Acc Chem Res* **2015**, *48* (8), 2380, https://doi.org/10.1021/acs.accounts.5b00157.

[9] G. Caserta, L. Pecqueur, A. Adamska-Venkatesh, C. Papini, S. Roy, V. Artero, M. Atta, E. Reijerse, W. Lubitz, M. Fontecave, Structural and functional characterization of the hydrogenase-maturation HydF protein, *Nat Chem Biol* **2017**, *13* (7), 779, https://doi.org/10.1038/nchembio.2385.

[10] C. Papini, C. Sommer, L. Pecqueur, D. Pramanik, S. Roy, E. J. Reijerse, F. Wittkamp, V. Artero, W. Lubitz, M. Fontecave, Bioinspired Artificial [FeFe]-Hydrogenase with a Synthetic H-Cluster, *ACS Catalysis* **2019**, *9* (5), 4495, https://doi.org/10.1021/acscatal.9b00540.

[11] S. T. Stripp, In Situ Infrared Spectroscopy for the Analysis of Gas-processing Metalloenzymes, *ACS Catalysis* **2021**, *11* (13), 7845, https://doi.org/10.1021/acscatal.1c00218.

[12] H. Li, T. B. Rauchfuss, Iron carbonyl sulfides, formaldehyde, and amines condense to give the proposed azadithiolate cofactor of the Fe-only hydrogenases, *J Am Chem Soc* **2002**, *124* (5), 726, https://doi.org/10.1021/ja016964n.
